# Mutual Placement of Isocyanide and Phosphine Ligands in Platinum(II) Complexes [PtHal_2_L^1^L^2^] (Hal = Cl, Br, I; L^1^,L^2^ = CNCy, PPh_3_) Leads to Highly-Efficient Photocatalysts for Hydrosilylation of Alkynes

**DOI:** 10.3390/molecules28237764

**Published:** 2023-11-24

**Authors:** Maria V. Kashina, Andrei A. Karcheuski, Mikhail A. Kinzhalov, Konstantin V. Luzyanin, Svetlana A. Katkova

**Affiliations:** 1 Institute of Chemistry, St. Petersburg University, 7/9 Universitetskaya Emb., Saint Petersburg 199034, Russiam.kinzhalov@spbu.ru (M.A.K.); 2 Department of Chemistry, University of Liverpool, Crown Street, Liverpool L69 7SD, UK

**Keywords:** isocyanide ligands, platinum(II) complexes, catalytic activity, hydrosilylation, photocatalysis

## Abstract

A series of platinum complexes featuring phosphine and isocyanide ligands [PtX_2_(PPh_3_)(CNCy)] (X = Cl, Br, and I) as well as their parent phosphine [PtX_2_(PPh_3_)_2_] and isocyanide [PtX_2_(CNCy)_2_] analogues have been prepared and evaluated as catalysts for the photocatalytic hydrosilylation of alkynes. Under violet light irradiation (λ_max_ = 400 nm), phosphine–isocyanides complexes [PtX_2_(PPh_3_)(CNCy)] gave high yields of silylated products (product yield up to 99%, TONs up to 1.98 × 10^3^). The blue light irradiation (λ_max_ = 450 nm) was more suitable for the parent phosphine complexes [PtX_2_(PPh_3_)_2_], which showed comparable efficiency (product yield up to 99%, TON up to 1.98 × 10^3^), while isocyanide complexes [PtX_2_(CNCy)_2_] were not active.

## 1. Introduction

Homogeneous catalysis by transition metal complexes has become an indispensable tool for organic chemists. Nowadays, it is almost unthinkable to consider synthetic strategies that do not rely on metal catalysis for C–C bond formation reactions, stereoselective synthesis, or regulation of redox processes. The prevalence of the late transition metal catalysts, mostly of the platinum group [1], *viz.* platinum, rhodium, iridium, and palladium, as well as their non-noble counterparts, *viz.* cobalt, iron, nickel, and manganese, can be rationalised by their expected two-electron transformations and intrinsic stability of metal centres under varying conditions. The catalytic activity of the transition metal catalysts can be tuned by the installation of appropriate ligands; the electron donor strength of the ligand affects the properties of the metal centre, such as its nucleophilicity and redox capability. The most common neutral ligands involved in transition metal catalysts are tertiary phosphines [2]. The phosphorous centre of this ligand is bound to transition metals through the σ-donation of its electron pair to a metal orbital and the π-backdonation occurring from filled metal orbitals to empty orbitals of the phosphine. The stability of phosphine complexes with platinum metals, relative ease in their preparation, and tunability justify their widespread application in the development of effective homogeneous catalysts [3].

Isocyanides are an alternative type of strong σ-donor, albeit weak π-acceptor ligands [4]. In fact, a wide range of reactions catalysed by isocyanide complexes have been discovered, i.e., addition to double and triple C-C bonds [5,6,7,8], functionalization of E–H [9,10,11] and E–E [12,13] to multiple bonds, alkyl and aryl halide activation [14,15], and electron transfer catalysis [16,17,18]. Isocyanides, in the same way as phosphines, have shown a tendency to form complexes with readily dissociated ligands [19,20], a useful starting point for the design of new catalysts.

While phosphines and isocyanides are primarily responsible for the tuning of the donor properties and stability of the metal centre, secondary halide ligands neutralise the charge of the metal centre and therefore stabilise the intermediates of the catalytic cycle [21,22]. For example, palladium Chugaev carbene complexes ([PdHal_2_(R^1^NCN(H)N(H)CNR^2^)]) bearing chlorides have shown superior catalytic efficiency to the bromide species as precatalysts for Suzuki–Miyaura cross-coupling [23]. To rationalise the selection and mutual influence of ancillary ligands on the catalytic properties of metal complexes, we have prepared a series of platinum’s dihalide complexes with varying sets of phosphine and isocyanide ligands and examined their catalytic activity for the hydrosilylation of alkynes under thermal and photocatalytic conditions.

## 2. Results and Discussion

### 2.1. Synthesys and Charactrization

As aliphatic isocyanides are known to have greater donor strength than their aromatic counterparts [24], cyclohexyl isocyanide was chosen for investigation, and triphenylphosphine was selected as one of the most commonly available tertiary phosphines. The series of new (**4**–**6**, **8**, **9**) and known (**1**–**3** [25,26], **7** [27]) platinum(II) *bis*phosphine, *bis*isocyanide, and phosphine/isocyanide complexes (Table 1) were prepared and characterised by CHN elemental analyses, high-resolution mass spectrometry (HR ESI^+^-MS), FT-IR, and ^1^H, ^13^C{^1^H}, ^31^P{^1^H}, and ^195^Pt{^1^H} NMR spectroscopy. The geometry of all the complexes in solution was assigned based on FT-IR and NMR spectroscopy (for detailed discussion, see Section S1 in the Supplementary Materials (SM)). All **1**–**9** are isomerically pure in the solution and the solid state.

The Pt–P one-bond spin–spin coupling constants (^1^*J*_Pt,P_) are dominated by the Fermi contact interaction of nuclei with *s-*orbital electrons and are used as an estimate of the bond strength (Table S2, see Supplementary Materials). Since the magnitude of the ^1^*J*_P,Pt_ is related to the *s*-character of the Pt–P bond, they ultimately give information about the effective nuclear charge on the platinum atom in the complexes [26,28]. The Pt–P bond strengths and ^1^*J*_Pt,P_ depend, among other things, on the nature of the other ligands, but the influence of the ligand in *trans* position is higher than that of the ligand in *cis* position [25,26]. In both series *cis-*[PtX_2_(PPh_3_)_2_] and *cis-*[PtX_2_(PPh_3_)(CNCy)], the ^1^*J*_Pt,P_ decreases in order of Cl > Br > I (Table S2) and matches the gradually declining *trans*-influence of these ligands [29]. The comparison of each pair *cis*-[PtX_2_(PPh_3_)_2_] and *cis-*[PtX_2_(PPh_3_)(CNCy)] with the same anionic ligands led to the conclusion that the ^1^*J*_Pt,P_ of **1**–**3** is bigger than the ^1^*J*_Pt,P_ of **4**–**6**, being consistent with the greater *σ*-donation and *π*-acceptance abilities of phosphines compared to isocyanides [30,31].

**Figure 1:**
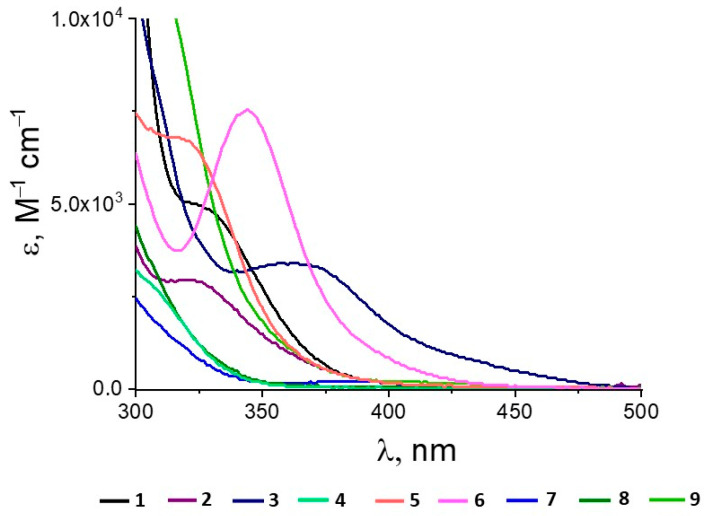
The fragment of UV/vis absorption spectra of complexes **1**–**9** in CH_2_Cl_2_ at RT (2 × 10^−5^ M).

The UV-vis absorption spectra of **1**–**9** were measured at ambient temperature in CH_2_Cl_2_ solution (Figure 1 and Section S6, Supplementary Materials). The prepared complexes are colourless (**1**, **4**, **7**), yellowish (**2**, **5**, **8**), or yellow (**3**, **6**, **9**) solids. The UV-vis spectra of **1**–**9** exhibit strong absorption bands below 300 nm [ε = (0.20–1.94) × 10^4^ M^−1^cm^−1^] that can be assigned to ligand-centred transitions of phosphine and/or isocyanide ligands [32]. The wide weak absorption bands in the 300–360 nm range with molar extinction coefficients [ε = (0.03–0.17) × 10^4^ M^−1^ cm^−1^] can be assigned the d → d, π → π*, n → π* and change-transfer transition arising from π electron interactions between the metal and ligands, which involve either a metal-to-ligand or ligand-to-metal electron transfer [33,34]. A study of the absorption spectra of the coloured complexes revealed that the long wavelength absorption maxima for the isocyanide derivatives **8**, **9** are lower in energy compared to the phosphine **2**, **3** and mixed ligand **5**, **6** analogues. According to the data in the series Cl > Br > I of complexes **1**–**9**, the energy is higher in complexes with iodine ligands **3** and **6** (Table S9, Figures S5–S7, Supplementary Materials) [33,35,36,37].

**Figure 2:**
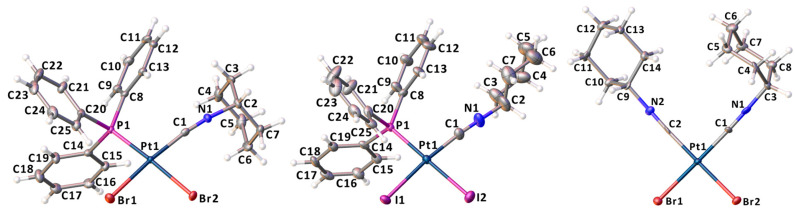
The structures of **5** (**left**), **6** (**centre**), and **8** (**right**). The thermal ellipsoids are shown at 50% probability.

The structures of **5**, **6,** and **8** were further confirmed by single-crystal X-ray diffraction (Figure 2, Section S2 in Supplementary Materials). All complexes have a square-planar coordination sphere (*τ*_4_ [38] = 0.02–0.05) with minor distortions. In **8,** the isocyanide ligands are in the *cis*-position, while related Pd^II^ species [PdBr_2_(CNR)_2_] are only *trans* [39,40,41]. As far as we know, the X-ray data for **8** are the first X-ray structures for [PtBr_2_(CNR)_2_]-type compounds. The fragments Pt–C≡N–C in all structures are nearly linear (Table S4, Supplementary Materials), indicating the weak π-acceptor effect of isocyanide ligands [42]. The metal–carbon bonds (1.916(7)–1.920(7) Å) in **8** are slightly longer than those in the chloride platinum species *cis-*[PtCl_2_(CNCy)_2_] (1.911(3) Å, 1.904(3) Å [27]), as bromide ligands have increased *trans*-influence compared to chlorides [43]. In **5**–**6**, the Pt1–X1 bonds, which are *trans* to CNCy ligands, are shorter than Pt1–X2 in *trans*-position to PPh_3_, indicating the greater trans-influence of PPh_3_ ligand vs. CNCy. The formally triple C≡N bond in the isocyanide ligands is 1.136(8)–1.149(8) Å long (Table S4, Supplementary Materials), which is the typical range for the CN triple bonds in the related isocyanide complexes, e.g., [MX_2_(CNR)_2_] (M = Pd, Pt; R = Xyl, Cy, Mes, C_6_H_4_-4-Hal (Hal = F, Cl, Br, I); X = Cl, Br; 1.135–1.155 Å) [44].

### 2.2. DFT Analysis of Ligand Properties

**Figure 3:**
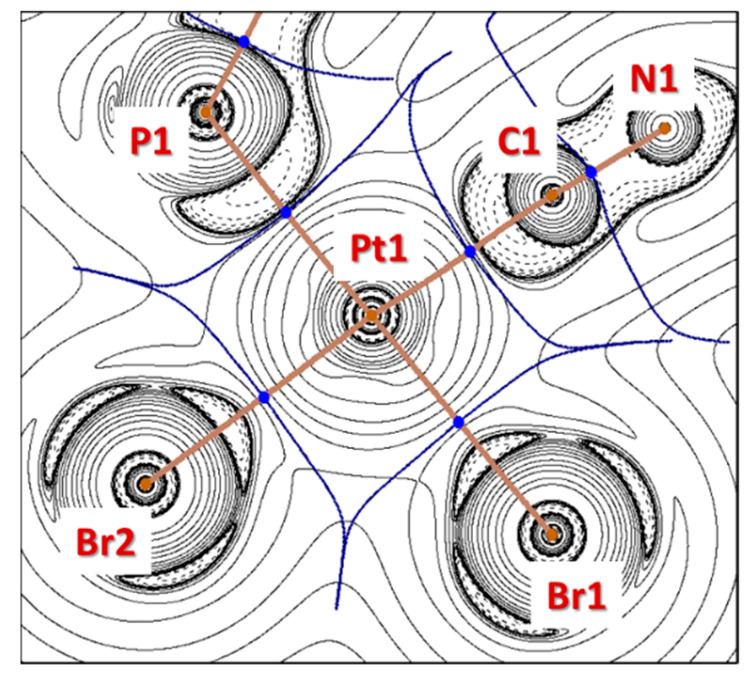
Visualisation of ∇^2^ρ(**r**) for coordination bonds in **5**. Bond critical points (3, −1) are shown in blue, nuclear critical points (3, −3) and bond paths—in pale brown.

Density functional theory (DFT) calculations were performed to determine if the bonding and electronic structure in the complexes could further explain the differences in the catalytical activity of **1**–**9** (Section S4, see Supplementary Materials). Geometry optimisations in the ground state resulted in structures in good agreement with experimental crystallographic data: changes in coordination bonds and angles were less than 3% (Table S6, see Supplementary Materials). Information on the binding of various metal complexes can be found through the topological analysis of the electron density distribution (AIM method) [45]. This approach was utilised in studying the nature of Pt–L coordination bonds in optimised equilibrium structures (Table 2 and Table S6, Figure 3 and Figure S4). The most important property to assign a bond is the presence of a bond critical point (BCP) between pairs of bonded atoms. The BCPs occur where the line of maximum density between two atoms, named the bond path, intersects the interatomic surfaces. The electron density value and its Laplacian at BCPs provide insights into the nature of interactions. As a rule, ρ(**r**) > 0.20 a.u. and ∇^2^ρ(**r**) < 0 indicate a covalent bond, while ρ(**r**) < 0.10 a.u. and ∇^2^ρ(**r**) > 0 indicate an ionic bond. More broadly, increasing values of ρ(**r**) and reductions in ∇^2^ρ(**r**) indicate increasing covalent character and stronger bonding interactions. The values of ρ(**r**) and ∇^2^ρ(**r**) parameters between platinum and the donor atom in these BCPs are typical for closed-shell dative interactions [46] (Table 2). Among **1**–**9**, the Pt–C bonds have larger values of ρ(**r**) than those of the Pt–P bonds, and for both series **1**–**3** and **4**–**6**, the values of ∇^2^ρ(**r**) of the Pt–P bonds remain unaltered (Table 2 and Figure 3). At the same time, in the mixed-ligand complexes **4**–**6**, the influence of the halogen’s type on the value ∇^2^ρ(**r**) Pt–C bonds can be seen with the maximum observed for **4** (0.171). However, all the BCP parameters for Pt–Hal bonds suggest no significant changes in ligand type variation.

Based on Espinosa’s criterion [47] for characterising interactions at the BCPs by analysing the ratio of the potential and kinetic energy densities, the |V(r)|/|G(r)| values of Pt–L BCPs are 1.41–1.82 (Table 2), indicating that these Pt–L bonding regions have a somewhat covalent character, which could well correspond to a dative bond type. In addition, the values for |V(**r**)|/G(**r**) for all Pt–P bonds are higher (1.69–1.82) than for the Pt–C bonds (1.53–1.56), indicating the Pt–P is more covalent.

The calculated Mayer bond orders (MBOs) [48] of the Pt–C bonds (0.90–1.02) are larger than those of the Pt–P bonds (0.65–0.89), demonstrating the weakened character of the latter. Meanwhile, Wiberg bond indexes at 1.28–1.43 [49] show that the Pt–C bonds have a partially double character through π-donation of electron density from the filled *d* orbitals of the platinum to the empty *p*_π_ orbitals of the C atoms.

### 2.3. Catalytic Study

**Scheme 1:**
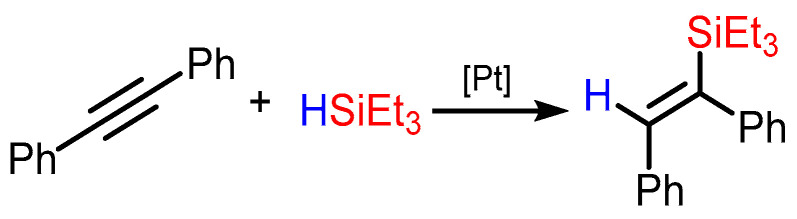
The model hydrosilylation reaction.

**Figure 4:**
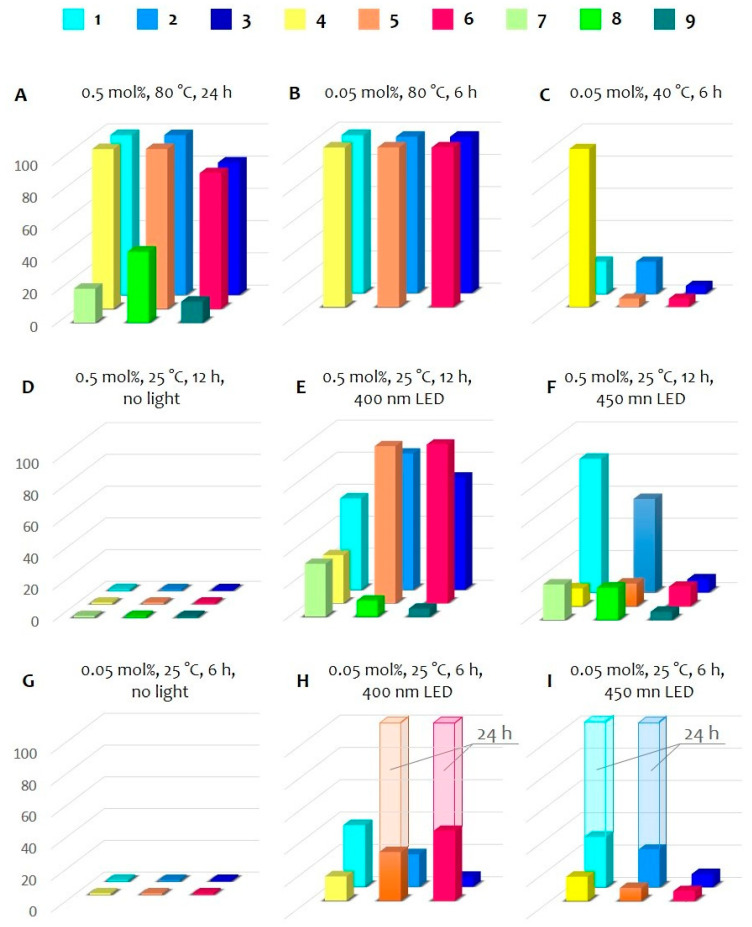
The screening of catalysts **1**–**9** in the hydrosilylation reaction (product yield, %). Conditions: 1,2-diphenylacetylene (5.0 × 10^−4^ mol), Et_3_SiH (7.5 × 10^−4^ mol), toluene (0.5 mL), selected catalyst (2.5 × 10^−7^ or 2.5 × 10^−6^ mol). The yield of 1,2-(diphenylvinyl)triethylsilane was determined by ^1^H NMR spectroscopy using 1,2-dimethoxyethane as internal standard; the product was obtained as a pure *E* isomer (*Z* < 5%) at 40–80 °C or as a mixture of *E*/*Z* isomers (*E* 75–85%) in light-induced catalytic reactions.

Prepared complexes **1**–**9** were evaluated as catalysts in the hydrosilylation of alkynes under both thermal and photocatalytic conditions (Table S8, Figure 4). As a model system, we have chosen the commonly used reaction of 1,2-diphenylacetylene with triethylsilane, producing 1,2-(diphenylvinyl)triethylsilane [10,50] (Scheme 1).

Screening the entire library of **1**–**9** as catalysts with loading of 0.5 mol% at 80 °C revealed large variations in catalytic activity as a function of catalyst structure, with yields of the 1,2-(diphenylvinyl)triethylsilane product ranging from 13% to 99% after 24 h (Figure 4A). Catalysts **1**–**6**, containing one (**4**–**6**) or two (**1**–**3**) PPh_3_ ligands, gave significantly higher yields than containing two isocyanides (**7**–**9**), possibly as a result of the greater donation ability of PPh_3_ compared to CNCy [51]. With lower catalyst loadings (0.05 mol%), all **1**–**6** showed elevated activity (97–99%, TONs 1.9–2.0 × 10^3^), and, surprisingly, no significant difference in activity was observed between phosphine complexes **1**–**3** and mixed ligand complexes **4**–**6** (Figure 4B).

At 40 °C, the application of 0.05 mol% of **1**–**6** showed differences between phosphine and mixed ligand phosphine-isocyanide complexes, and under these conditions, the mixed ligand complex **4** showed superior catalytic activity (Figure 4C). This example illustrates the complementary role of the isocyanide and phosphine ligands. The *bis-*isocyanide complex itself is not capable of catalysing the hydrosilylation, probably because the isocyanide is not a sufficient donor and cannot facilitate the oxidative addition step [52,53]. However, the *bis*-phosphine complex exhibits weaker catalytic activity as well, presumably due to excessive shielding of the metal centre by bulky tertiary phosphine ligands [54]. The combination of the phosphine and isocyanide ligands on one platinum centre represents an optimal compromise between nucleophilicity and spatial availability of the metal centre, leading to superior catalytic efficiency.

According to the data along the series Cl–Br–I, the complexes with chloride and bromide ligands were more effective in hydrosilylation than the iodide complexes (Table S8, Figure 4). To the best of our knowledge, only one example of a comparison of catalytic activity for different platinum(II) halides has been described beforehand. In [55,56,57], Pt^II^-NHC complexes featuring ancillary iodide ligands showed lower catalytic activity in the hydrosilylation process when compared to the bromide species; no comparison to chloride analogues has been provided.

Visible light-induced catalytic reactions have become a powerful trend in organic synthesis [58,59,60,61]. Extending this strategy to platinum(II)–catalysed hydrosilylation under visible light irradiation [10] brings up the photocatalytic system in which a transition metal catalyst serves as both a photo-absorber and a catalysis-enabling species [62,63]. Despite the fact that all **1**–**9** have weak absorption bands in the visible light region, the species were evaluated as hydrosilylation catalysts under visible light irradiation [50].

The initial test of catalysts **1**–**9** (0.5 mol%) showed that the complexes **1**–**6** containing phosphine ligands exhibit superior efficiencies as compared to *bis*isocyanide species **7**–**9** under irradiation with violet (λ = 400 nm) and blue (λ = 450 nm) light during 12 h (Figure 4D,E). The blue irradiation is more convenient for the phosphine complexes **1**–**3** (Figure 4D), whereas the violet irradiation is more convenient for the mixed ligand’s phosphine-isocyanide complexes **4**–**6** (Figure 3E). Under blue irradiation (λ = 450 nm), catalyst loading of 0.05 mol% resulted in low to moderate activity (6–31% product yields after 6 h, TON = 1.2–6.2 × 10^2^/1.2–103.3); complexes **1** and **2** were the most active with 31% (TON = 6.2 × 10^2^) and 23% (TON = 4.6 × 10^2^) product yields (Table S8), respectively. Increasing the reaction time to 24 h led to a nearly quantitative conversion of the starting 1,2-diphenylacetylene to the respective silylated product. The findings of this study agree with previous studies indicating the possibility of photodissociation of PPh_3_ ligand under visible light irradiation [63,64].

The observed photocatalytic activity is not aligned directly with the photophysical properties of the complexes under study. Within the series of *bis*phosphine complexes **1**–**6**, there is a noticeable increase in light absorption in the visible region on going from chlorine to bromine to iodine, while the catalytic activity of respective complexes decreases. The efficiency of **1**–**6** is lower compared to the most efficient protocols for light-induced hydrosilylation described previously, which were based on mixed-ligand Pt^II^-diaminocarbene/isocyanide complexes [10,50].

## 3. Materials and Methods

### 3.1. Instruments and Reagents

Solvents and organic and inorganic reagents were obtained from commercial sources and used as received. The complexes **1**–**3** and **7** were obtained using known methods [25,26,27,65]. C, H, and N elemental analyses were carried out on a Euro EA 3028 HT CHNS analyzer. High-resolution mass spectra were acquired on a Bruker micrOTOF spectrometer equipped with an electrospray ionisation (ESI) source. MeOH was used as the solvent. The instrument was operated in positive ion modes using a *m*/*z* range of 50–3000. The capillary voltage of the ion source was set at −4500 V (ESI^+^) and the capillary exit at +(70–150) V. The nebulizer gas pressure was 0.4 bar, and the drying gas flow was 4.0 L/min. The most intensive peak in the isotopic pattern is reported. Infrared spectra were recorded on the Shimadzu IRAffinity-1 FTIR instrument (4000–400 cm^−1^, resolution 2 cm^−1^) in KBr pellets. NMR spectra were recorded on a Bruker AVANCE III 400 spectrometer in CDCl_3_ at ambient temperature (at 400, 100, 162, and 86 MHz for ^1^H, ^13^C, ^31^P, and ^195^Pt NMR, respectively). Chemical shifts are given in δ-values [ppm] referenced to the residual signals of undertreated solvent (CHCl_3_): δ 7.26 (^1^H) and 77.2 (^13^C). ^1^H and ^13^C NMR data assignment for **4**–**7** was achieved by using 2D (^1^H,^1^H-COSY, ^1^H,^1^H-NOESY, ^1^H,^13^C-HMQC/HSQC and ^1^H,^13^C-HMBC) NMR correlation experiments. The UV/vis absorption spectra in CH_2_Cl_2_ solution were recorded on a Shimadzu UV-2500 (Shimadzu Corporation, Tokyo, Japan) spectrophotometer in a quartz cuvette with l = 1.0 mm and the complex’s concentration of 0.03 mM.

**X-ray Diffraction Study.** X-ray diffraction studies of single crystals of complexes **4**, **6**, and **7**. Single crystals for X-ray diffraction experiments were grown by the slow evaporation of the respective compounds in the CH_2_Cl_2_/Et_2_O mixture under air at room temperature. A single-crystal X-ray diffraction experiment was carried out on a SuperNova, single source at offset/far, HyPix3000 diffractometer with monochromated CuKα radiation. The crystal was kept at 100(2) K during data collection. The structures were resolved by ShelXT [66] (a structure solution programme using intrinsic phasing) and refined by means of the ShelXL [67] programme incorporated into the OLEX2 program package [68]. The crystallographic details are summarised in Table S1. Empirical absorption correction was applied in the CrysAlisPro (Agilent Technologies, Yarnton, UK, 2012) program complex using the spherical harmonics implemented in the SCALE3 ABSPACK scaling algorithm. The crystal data, data collection parameters, and structure refinement data for **4**, **6**, and **7** are given in Table S3, and the plots and selected bond lengths and angles are given in Table S4 CCCDC numbers 1811734 (**4**), 1839000 (**6**), and 1839001 (**7**) contain the supplementary crystallographic data for this paper. These data can be obtained free of charge from the Cambridge Crystallographic Data Centre via www.ccdc.cam.ac.uk/data_request/cif (access date: 23 October 2023).

**Computational Details.** The geometry of **1**–**9** were optimised at density functional theory (DFT) calculations at the M06 [69]/Def2SVP [70,71] level with the help of Gaussian-09 [72]. The single-point calculations for optimised structures have been carried out using the PBE0 [73]-D3/def2-TZVP [74] level of theory with also help of the Gaussian-09 programme package. The topological analysis of the electron density distribution with the help of the atoms in molecules (QTAIM) method developed by Bader [45], MBO [75], and WI [49] analysis been performed using the Multiwfn programme (version 3.7) [76]. The Cartesian atomic coordinates for all model structures are presented in Table S7.

### 3.2. Synthesis and Characterization

**Synthesis of ***cis**-*****[PtCl_2_(CNCy)(PPh_3_)]** (**4**). Solution of CyNC (39 mg, 0.27 mmol) in 2 mL DCE was sequentially added to a solution of [PtCl_2_(NCEt)_2_] (100 mg, 0.27 mmol) in 20 mL DCE, and then solid PPh_3_ (71 mg, 0.27 mmol) was inserted. The reaction mixture was heated for 2 h under reflux and then reduced to 2 mL with evaporation. The white precipitate of *cis-*[PtCl_2_(CNCy)(PPh_3_)] was formed after being diluted with Et_2_O (5 mL), separated with centrifugation, washed with two 5-mL portions of Et_2_O, and dried in air. Yield: 87% (149 mg). Anal. Calcd. for C_25_H_26_NCl_2_PPt: C, 47.10; H, 4.11; N, 2.20, found: C, 46.88; H, 4.09; N, 2.18. ESI^+^-MS, *m*/*z*: calcd. for C_25_H_26_Cl_2_NNaPPt^+^ 660.0708, found 660.0712 [M + Na]^+^. IR (ν, cm^−1^): 2931 ν*_s_* (C–H, Cy), 2857 ν*_s_* (C–H, Cy), 2227 ν_s_ (C≡N). ^1^H NMR (δ, ppm): 1.16–1.78 m (10H, CH, Cy), 3.32–3.37 m (1H, CH, Cy), 7.42–7.53 m (9H, Ar–H), 7.69–7.75 m (6H, Ar–H). ^13^C{^1^H, ^31^P} NMR (δ, ppm): 22.5 (CH_2_, Cy), 24.6 (CH_2_, Cy), 31.3 (CH_2_, Cy), 55.0 (CH, Cy), 128.6 (*o–*CH from Ph), 128.7 (*ipso*–C from Ph), 131.7 (*p*–CH from Ph), 134.6 (*m*–CH from Ph); the C_C≡N_ resonances were not detected. ^31^P{^1^H} NMR (δ, ppm): 8.4 (*J*_P,Pt_ = 3416 Hz). ^195^Pt{^1^H} NMR (δ, ppm): −4118 (*J*_Pt,P_ = 3416, *J*_Pt,N_ = 112 Hz).

**Synthesis of ***cis**-*****[PtX_2_(CNCy)(PPh_3_)] (X = Br (5), I (6)**. Solid KBr (30 mg, 0.25 mmol) or KI (42 mg, 0.25 mmol) was added to a solution of **2** (64 mg, 0.10 mmol) in acetone (10 mL) at 20–25 °C. The reaction mixture was stirred at RT for ca. 4 d (for KBr) or 2 h (for KI). The yellow (for KBr) or dark red (for KI) suspension formed was evaporated at 40–45 °C, and the product was extracted with twenty 5-mL portions of CH_2_Cl_2_. The resulting bright yellow solution was evaporated to a volume of 2 mL, and a sediment was formed after the addition of Et_2_O to the residue, which was washed with two 5-mL portions of Et_2_O, and then dried in vacuo at RT.

*cis**-*****[PtBr_2_(CNCy)(PPh_3_)] (5).** Yield: 91% (66 mg). Anal. Calcd. for C_25_H_26_NBr_2_PtP: C, 41.34; H, 3.61; N, 1.93 found: C, 41.02; H, 3.62; N, 1.94. ESI^+^-MS, *m*/*z*: calcd. for C_25_H_26_Br_2_NNaPPt^+^ 749.9689, found 749.9691 [M + Na]^+^. IR (ν, cm^−1^): 3087 ν*_w_*(C–H, Ar), 3064 *w*, 3046 *w*, 2227 ν*_s_* (C≡N). ^1^H NMR (δ, ppm): 1.18–1.44 m (6H, CH, Cy), 1.52–1.68 m (4H, CH, Cy), 3.29–3.41 m (1H, CH, Cy), 7.43–7.57 m (9H, Ph), 7.71–7.81 m (6H, Ph). ^13^C{^1^H, ^31^P} NMR (δ, ppm): 22.5 (CH_2_, Cy), 24.6 (CH_2_, Cy), 31.3 (CH_2_, Cy), 55.0 (CH, Cy), 128.4 (*o–*CH from Ph), 128.5 (*ipso*–C from Ph), 129.2 (C≡N), 131.5 (*p–*CH from Ph), 134.6 (*m–*CH from Ph). ^31^P{^1^H} NMR (δ, ppm): 8.5 (*J*_P,Pt_ = 3358 Hz). ^195^Pt{^1^H} NMR (δ, ppm): −4394 (*J*_Pt,P_ = 3358 Hz, *J*_Pt,N_ = 109 Hz).

*cis**-*****[PtI_2_(CNCy)(PPh_3_)] (6).** Yield: 85% (70 mg). Anal. Calcd. for C_25_H_26_NI_2_PtP: C, 36.60; H, 3.19; N, 1.71 found: C, 37.31; H, 3.21; N, 1.68. For C_25_H_26_I_2_NNaPPt^+^ 842.9435, found 842.9404 [M + Na]^+^. IR (ν, cm^−1^): 3087 ν*_w_*(C–H, Ar), 3064 (*w*), 3046 (*w*), 2239 ν_s_ (C≡N). ^1^H NMR (δ, ppm): 1.20–1.39 m (6H, CH, Cy), 1.51–1.66 m (4H, CH, Cy), 3.25–3.36 m (1H, CH, Cy), 7.41–7.51 m (9H, Ph), 7.70–7.76 m (6H, Ph). ^13^C{^1^H, ^31^P} NMR (δ, ppm): 22.4 (CH_2_, Cy), 24.6 (CH_2_, Cy), 31.0 (CH_2_, Cy), 54.8 (CH, Cy), 128.3 (o–CH from Ph), 130.2 (ipso–C from Ph), 131.4 (p–CH from Ph), 134.7 (m–CH from Ph); the C_C≡N_ resonances were not detected. ^31^P{^1^H} NMR (δ, ppm): 7.6 (*J*_P,Pt_ = 3216 Hz). ^195^Pt{^1^H} NMR (δ, ppm): −4985 (*J*_Pt,P_ = 3216, *J*_Pt,N_ = 102 Hz).

**Synthesis of [PtX_2_(CNCy)_2_] (X = Br, I)** (**8–9**). Solid KBr (60 mg, 0.5 mmol) or KI (84 mg, 0.5 mmol) was added to a solution of [PtCl_2_(CNCy)_2_] (75 mg, 0.20 mmol) in acetone (20 mL) at 20–25 °C. The reaction mixture was stirred at RT for ca. 4 days. The formed suspension was evaporated at 40–45 °C, and the product was extracted with twenty 5-mL portions of CH_2_Cl_2_. The resulting bright yellow solution was evaporated to a volume of 2 mL, and a sediment was formed after the addition of Et_2_O to the residue, which was washed with two 5-mL portions of Et_2_O, and then dried in vacuo at RT.

*cis-***[PtBr_2_(CNCy)_2_] (8)**. Yield: 90% (67 mg). Anal. Calcd. for C_14_H_22_N_2_Br_2_Pt: C, 29.33; H, 3.87; N, 4.89, found: C, 28.98; H, 3.85; N, 4.63. ESI^+^-MS, *m*/*z*: calcd. for C_14_H_22_Br_2_N_2_NaPt^+^ 596.9670, found 596.9660 [M + Na]^+^. IR (ν, cm^−1^): 2261 ν_s_ (C≡N), 2233 ν_s_ (C≡N). ^1^H NMR (δ, ppm): 1.43–1.98 (m, 20H, CH_2_, Cy), 4.00–4.17 (m, 2H, CH, Cy). ^13^C{^1^H} NMR (δ, ppm): 22.5 (CH_2_, Cy), 24.7 (CH_2_, Cy), 31.7 (CH_2_, Cy), 55.7 (CH, Cy), 106.6 (C≡N, *J*_C,N_ = 26.6, *J*_C,Pt_ = 1620 Hz). ^195^Pt{^1^H} NMR (δ, ppm): −4107 (*J*_Pt,N_ = 100 Hz).

*trans*-**[PtI_2_(CNCy)_2_] (9)**. Yield: 82% (109 mg). ESI^+^-MS, *m*/*z*: calcd. for C_14_H_22_I_2_N_2_NaPt^+^ 689.9413, found 689.9397 [M + Na]^+^. Anal. Calcd. for C_14_H_22_N_2_I_2_Pt: C, 25.20, H, 3.32, N, 4.20, found: C, 24.95; H, 3.33; N, 4.56. IR (KBr, selected bands, cm^−1^): 2220 ν_s_ (C≡N). ^1^H NMR (δ, ppm): 1.50–2.02 (m, 20H, CH_2_, Cy), 4.02–4.14 (m, 2H, CH, Cy). ^13^C{^1^H} NMR (δ, ppm): 22.3 (CH_2_, Cy), 24.8 (CH_2_, Cy), 31.7 (CH_2_, Cy), 55.1 (CH, Cy), 117.2 (C≡N, *J*_C,N_ = 24.7 Hz). ^195^Pt{^1^H} NMR (δ, ppm): −5408 (*J*_Pt,N_ = 87 Hz).

### 3.3. Catalytic Tests

General procedure for the catalytic hydrosilylation of alkynes with hydrosilanes (specific conditions are provided in Table S8). The solution of the selected catalyst in CH_2_Cl_2_ (0.1 mL, 5.0 × 10^−6^ M) was placed in the 5 mm tubes, and the solvent was evaporated to dryness under a stream of dinitrogen. 1,2-diphenylacetylene (5.0 × 10^−4^ mol), Et_3_SiH (7.5 × 10^−4^ mol), and toluene (2 mL) were added to the tube. The tube was closed with a septum, kept at 40–80 °C or under LED irritation with 400/450 nm LEDs (3 × 2.5 W, the LEDs were placed at 2 cm distance from the tube) for 6–24 h. The contents of the tube were poured over silica–gel and extracted with hexane (5 mL). Extracts were evaporated under reduced pressure, and the crude product was subsequently dissolved in 0.6 mL of CDCl_3_ with 1,2-dimethoxyethane (1.0 equiv, used as an NMR internal standard) added, and then analysed by ^1^H and ^13^C NMR spectroscopy. The isomeric content was determined on the basis of the analysis and matching of ^1^H and ^13^C chemical shifts for products against authentic samples of (*E*)-1,2-(diphenylvinyl)triethylsilane [77] and (*S*)-1,2-(diphenylvinyl)triethylsilane [78]. Quantifications were undertaken upon integration of the selected peaks of the product against peaks of 1,2-dimethoxyethane.

## 4. Conclusions

A series of platinum(II) complexes [PtX_2_L^1^L^2^] (**1**–**9**) bearing neutral ligands L (L^1^ = L^2^ = PPh_3_; L^1^ = PPh_3_, L^2^ = CNCy; L^1^ = L^2^ = CNCy) and varying halide ligands (X = Cl, Br, I) were prepared and evaluated as catalysts in homogeneous hydrosilylation of alkynes with hydrosilanes. All phosphine species **1**–**6** demonstrated high catalytic activity at 80 °C with a typical catalyst loading of 0.05 mol%, allowing the transformation of 1,2-diphenylacetylene and triethylsilane to the respective 1,2-(diphenylvinyl)triethylsilane with yields up to 98% (TON = 1.98 × 10^3^). Only *cis-*[PtCl_2_(PPh_3_)(CNCy)] (**4**) was catalytically active at 40 °C.

All **1**–**9** absorb light up to 400 nm, making these complexes suitable for visible-light-activated reactions. The absorption maxima of **1**–**9** have been red-shifted in order *bis*phosphine > phosphine/isocyanide > *bis*isocyanide complexes and Cl > Br > I. The blue light irradiation (λ_max_ = 450 nm) is more suitable for phosphine complexes **1**–**3** (up to 99% product yield, TON up to 1.98 × 10^3^), while phosphine–isocyanide species **4**–**6** better operated under violet light (λ_max_ = 400 nm, up to 99% product yield, TON up to 1.98 × 10^3^). The isocyanide complexes **7**–**9** demonstrate low catalytic activity under all conditions. Future studies aimed at understanding the mechanism of the photocatalytic action of the phosphine and isocyanide complexes and widening the scope of their catalytic applications are currently being expanded within our group.

## Figures and Tables

**Table 1:** The numbering scheme of the prepared platinum(II) complexes used as catalysts in the hydrosilylation of alkynes.

[PtX_2_L^1^L^2^]	L^1^ = L^2^ = PPh_3_	L^1^ = PPh_3_, L^2^ = CNCy	L^1^ = L^2^ = CNCy
X = Cl	*cis-*[PtCl_2_(PPh_3_)_2_] (**1**)	*cis-*[PtCl_2_(PPh_3_)(CNCy)] (**4**)	*cis-*[PtCl_2_(CNCy)_2_] (**7**)
X = Br	*cis-*[PtBr_2_(PPh_3_)_2_] (**2**)	*cis-*[PtBr_2_(PPh_3_)(CNCy)] (**5**)	*cis-*[PtBr_2_(CNCy)_2_] (**8**)
X = I	*trans-*[PtI_2_(PPh_3_)_2_] (**3**)	*cis-*[PtI_2_(PPh_3_)(CNCy)] (**6**)	*trans-*[PtI_2_(CNCy)_2_] (**9**)

**Table 2:** Bond orders and QTAIM of complexes **1**–**9**, where values of the density of all electrons—ρ(**r**), Laplacian of electron density—∇^2^ρ(**r**), energy density—H_b_, potential energy density—V(**r**), Lagrangian kinetic energy—G(**r**) (a.u.), MBO—Mayer bond order, and WI—Wiberg bond indices.

Bond	MBO	WI	ρ	G(r)	V(r)	H(r)	∇^2^ρ(r))	|V(r)|/G(r)
**1**								
Pt1–P1	0.7300	1.066	0.116	0.071	−0.127	−0.056	0.059	1.79
Pt1–P2	0.6888	1.026	0.111	0.066	−0.118	−0.052	0.061	1.79
Pt1–Cl1	0.6800	1.093	0.087	0.071	−0.100	−0.029	0.173	1.41
Pt1–Cl2	0.7279	1.185	0.092	0.075	−0.106	−0.032	0.176	1.41
**2**								
Pt1–P1	0.7118	1.031	0.112	0.068	−0.121	−0.053	0.064	1.78
Pt1–P2	0.6971	0.997	0.107	0.064	−0.112	−0.048	0.066	1.75
Pt1–Br1	0.6591	1.147	0.078	0.052	−0.076	−0.023	0.118	1.46
Pt1–Br2	0.6610	1.227	0.082	0.055	−0.080	−0.026	0.118	1.45
**3**								
Pt1–P1	0.6871	0.998	0.108	0.065	−0.114	−0.048	0.069	1.75
Pt1–P2	0.6547	0.962	0.102	0.062	−0.105	−0.044	0.072	1.69
Pt1–I1	0.6680	1.131	0.066	0.033	−0.051	−0.018	0.062	1.55
Pt1–I2	0.6603	1.211	0.070	0.035	−0.055	−0.020	0.061	1.57
**4**								
Pt1–P1	0.8866	1.028	0.116	0.068	−0.124	−0.056	0.049	1.82
Pt1–C1	1.0264	1.428	0.171	0.185	−0.289	−0.105	0.335	1.56
Pt1–Cl1	0.6986	1.151	0.089	0.071	−0.101	−0.030	0.169	1.42
Pt1–Cl2	0.6711	1.146	0.091	0.076	−0.107	−0.031	0.181	1.41
**5**								
Pt1–P1	0.8682	1.004	0.113	0.066	−0.120	−0.053	0.053	1.81
Pt1–C1	0.9955	1.394	0.168	0.179	−0.279	−0.100	0.327	1.56
Pt1–Br1	0.6763	1.187	0.080	0.052	−0.077	−0.024	0.114	1.48
Pt1–Br2	0.6832	1.202	0.081	0.056	−0.082	−0.026	0.121	1.46
**6**								
Pt1–P1	0.8141	0.983	0.110	0.064	−0.115	−0.051	0.057	1.80
Pt1–C1	0.9355	1.347	0.161	0.171	−0.264	−0.093	0.322	1.54
Pt1–I1	0.6811	1.192	0.069	0.035	−0.056	−0.020	0.061	1.60
Pt1–I2	0.6686	1.166	0.067	0.034	−0.053	−0.019	0.061	1.56
**7**								
Pt1–C1	0.9703	1.343	0.164	0.172	−0.269	−0.097	0.314	1.56
Pt1–C2	0.9572	1.335	0.162	0.171	−0.266	−0.095	0.318	1.56
Pt1–Cl1	0.6820	1.163	0.091	0.074	−0.106	−0.032	0.171	1.43
Pt1–Cl2	0.7101	1.180	0.093	0.075	−0.108	−0.033	0.171	1.44
**8**								
Pt1–C1	0.9411	1.310	0.160	0.167	−0.259	−0.092	0.314	1.55
Pt1–C2	0.9369	1.310	0.159	0.167	−0.259	−0.091	0.316	1.55
Pt1–Br1	0.6984	1.220	0.083	0.055	−0.082	−0.027	0.113	1.49
Pt1–Br2	0.7097	1.220	0.083	0.055	−0.081	−0.027	0.113	1.47
**9**								
Pt1–C1	0.9001	1.282	0.155	0.163	−0.250	−0.087	0.315	1.53
Pt1–C2	0.9063	1.285	0.156	0.164	−0.251	−0.087	0.316	1.53
Pt1–I1	0.7198	1.215	0.071	0.035	−0.056	−0.021	0.054	1.60
Pt1–I2	0.7150	1.202	0.070	0.035	−0.056	−0.021	0.055	1.60

## Data Availability

The data that support the findings of this study are available upon reasonable request from the authors.

## Supplementary Materials

The following supporting information can be downloaded at: https://www.mdpi.com/article/10.3390/molecules28237764/s1. Figure S1. The structures of complex **5**. The thermal ellipsoids are shown at 50% probability. Figure S2. The structures of complex 6. The thermal ellipsoids are shown at 50% probability. Figure S3. The structures of complex 8. The thermal ellipsoids are shown at 50% probability. Figure S4. View of optimised structures of complexes 1–9. Figure S5. UV/vis absorption spectra of complexes 1–3 in CH_2_Cl_2_ at RT (2 × 10^−5^ M). Figure S6. UV/vis absorption spectra of complexes 4–6 in CH_2_Cl_2_ at RT (2 × 10^−5^ M). Figure S7. UV/vis absorption spectra of complexes 7–9 in CH_2_Cl_2_ at RT (2 × 10^−5^ M). Figure S8. The ^1^H NMR spectra of [PtCl_2_(CNCy)(PPh_3_)_2_] 4. Figure S9. The ^13^C{^1^H, ^31^P} NMR spectra of [PtCl_2_(CNCy)(PPh_3_)_2_] 4. Figure S10. The ^31^P{^1^H} NMR spectra of [PtCl_2_(CNCy)(PPh_3_)_2_] 4. Figure S11. The ^195^Pt{^1^H} NMR spectra of [PtCl_2_(CNCy)(PPh_3_)_2_] 4. Figure S12. The ^1^H NMR spectra of [PtBr_2_(CNCy)(PPh_3_)_2_] 5. Figure S13. The ^13^C{^1^H, ^31^P} NMR spectra of [PtBr_2_(CNCy)(PPh_3_)_2_] 5. Figure S14. The ^31^P{^1^H} NMR spectra of [PtBr_2_(CNCy)(PPh_3_)_2_] 5. Figure S15. The ^195^Pt{^1^H} NMR spectra of [PtBr_2_(CNCy)(PPh_3_)_2_] 5. Figure S16. The ^1^H NMR spectra of [PtI_2_(CNCy)(PPh_3_)_2_] 6. Figure S17. The ^13^C{^1^H, ^31^P} NMR spectra of [PtI_2_(CNCy)(PPh_3_)_2_] 6. Figure S18. The ^31^P{^1^H} NMR spectra of [PtI_2_(CNCy)(PPh_3_)_2_] 6. Figure S19. The ^195^Pt{^1^H} NMR spectra of [PtI_2_(CNCy)(PPh_3_)_2_] 6. Figure S20. The ^195^Pt{^1^H} NMR spectra of [PtCl_2_(CNCy)_2_] 7. Figure S21. The ^1^H NMR spectra of [PtBr_2_(CNCy)_2_] 8. Figure S22. The ^13^C{^1^H} NMR spectra of [PtBr_2_(CNCy)_2_] 8. Figure S23. The ^195^Pt{^1^H} NMR spectra of [PtBr_2_(CNCy)_2_] 8. Figure S24. The ^1^H NMR spectra of [PtI_2_(CNCy)_2_] 9. Figure S25. The ^13^C{^1^H} NMR spectra of [PtI_2_(CNCy)_2_] 9. Figure S26. The ^195^Pt{^1^H} NMR spectra of [PtI_2_(CNCy)_2_] 9. Figure S27. The FTIR spectra of [PtCl_2_(CNCy)(PPh_3_)_2_] 4. Figure S28. The FTIR spectra of [PtBr_2_(CNCy)(PPh_3_)_2_] 5. Figure S29. The FTIR spectra of [PtI_2_(CNCy)(PPh_3_)_2_] 6. Figure S30. The FTIR spectra of [PtBr_2_(CNCy)_2_] 8. Figure S31. The mass spectra of [PtCl_2_(CNCy)(PPh_3_)_2_] 4. Figure S32. The mass spectra of [PtBr_2_(CNCy)(PPh_3_)_2_] 5. Figure S33. The mass spectra of [PtI_2_(CNCy)(PPh_3_)_2_] 6. Figure S34. The mass spectra of [PtBr_2_(CNCy)_2_] 8. Figure S35. The mass spectra of [PtI_2_(CNCy)_2_] 9. Table S1. The numbering scheme of prepared platinum(II) complexes used as catalysts in the hydrosilylation of alkynes. Table S2. ^31^P and ^1^*J*_P,Pt_ NMR data for [PtX_2_(PPh_3_)_2_] and *cis-*[PtX_2_(PPh_3_)(CNCy)]. Table S3. Crystal data and structure refinement for 5, 6, 8. Table S4. Selected bond lengths (Å) and angles (°) for 5, 6, 8. There are two independent molecules in the unit cell in structure 6, which are denoted by A and B letters. Table S5. Structures of [PtHal_2_(CNR)(X)] (X = CNR, PR’_3_) observed by CCDC search. Table S6. Bond orders and QTAIM of complexes 1–9, where values of the density of all electrons—ρ(r), Laplacian of electron density—∇^2^ρ(r), energy density—H_b_, potential energy density—V(r), Lagrangian kinetic energy—G(r) (a.u.), MBO—Mayer bond order, and WI—Wiberg bond indices. Table S7. Cartesian atomic coordinates for optimised structures. Table S8. Photocatalytic activity of complexes observed in this work in the model hydrosilylation reaction. Table S9. UV-Vis absorption data for 1–9. References [11,25,26,27,40,41,44,65,79,80,81,82,83,84,85,86,87,88,89,90,91,92,93,94,95,96,97,98,99,100,101] are cited in the Supplementary Materials.
