# Supplementary material for: Mutual Placement of Isocyanide and Phosphine Ligands in Platinum(II) Complexes [PtHal2L1L2] (Hal = Cl, Br, I; L1,L2 = CNCy, PPh3) Leads to Highly-Efficient Photocatalysts for Hydrosilylation of Alkynes
Source: Molecules. 2023 Nov 24;28(23):7764. doi: 10.3390/molecules28237764 (PMC10708205; doi:10.3390/molecules28237764)
Supplement: Supplementary file 1 [file molecules-28-07764-s001.zip › molecules-2712138-supplementary.pdf]

Supplementary Materials

Mutual Placement of Isocyanide and Phosphine Ligands in Platinum(II) Complexes [$PtHal_2L^1L^2$] (Hal = Cl, Br, I; L^1,L^2 = CNCy, PPh_3) Leads to Highly-Efficient Photocatalysts for Hydrosilylation of Alkynes

Maria V. Kashina [1], Andrei A. Karcheuski [1], Mikhail A. Kinzhalov [1], Konstantin V. Luzyanin [2,*] and Svetlana A. Katkova [1,*]

[1] Institute of Chemistry, St. Petersburg University, 7/9 Universitetskaya Emb., Saint Petersburg 199034, m.kinzhalov@spbu.ru (M.A.K.)

[2] Department of Chemistry, University of Liverpool, Crown Street, Liverpool L69 7SD, UK

* Correspondence: konstantin.luzyanin@liverpool.ac.uk (K.V.L.); s.katkova@spbu.ru (S.A.K.)

Table of content

S1. Preparation and Characterization. 3
S2. X-ray diffraction studies 6
S3. CCDC search. 10
S4. Theoretical studies 11
S5. Catalytic investigations 25
S6 UV/vis absorption spectra 27
S7. NMR spectra 29
S8. FTIR spectra 39
S9. Mass spectra 41
References 44

S1. Preparation and Characterization.

The platinum platinum(II) *bis*isocyanide and *bis*phosphine complexes (Table S1) were synthesized according to the previously published procedure [1-4]. While it is known that [$PtCl_2(PPh_3)_2$], [$PtBr_2(PPh_3)_2$] and [$PtI_2(PPh_3)_2$] can be prepared in the *cis* and *trans* isomers [3, 4], our investigation yielded the thermodynamic products *cis*-[$PtCl_2(PPh_3)_2$] (**1**), *cis*-[$PtBr_2(PPh_3)_2$] (**2**), and *trans*-[$PtI_2(PPh_3)_2$] (**3**) through the methods previously described [3, 4]. As expected, the adding an equal amount of phosphine and isocyanide to a solution of *cis*-[$PtCl_2(NCEt)_2$] in 1,2-dichloroethane led to the formation of mixed-ligand phosphine-isocyanide complex [$PtCl_2(CNCy)(PPh_3)$] (**4**) in yield of 87% as a single product. Additionally, **4** can be synthesized by combining equivalent quantities of **1** and **7** in CH_2Cl_2 at room temperature with a quantitative yield. The subsequent treatment of **4** with KBr or KI in acetone resulted in the formation of species **5** and **6**, respectively, in excellent yields (91% for **5** and 85% for **6**). The [$PtCl_2(PR'_3)(CNR)$] type complexes, in both solid phase and solution, display thermodynamic resistance towards *cis* to *trans* isomerisation and ligand disproportionation [5-7], therefore, the *cis* configuration was assigned to the complex **4.**

Table S1. The numbering scheme of prepared platinum(II) complexes used as catalysts in the hydrosilylation of alkynes.

**[$PtX_2L^1L^2$]**	**$L^1 = L^2 = PPh_3$**	**$L^1 = PPh_3, L^2 = CNCy$**	**$L^1 = L^2 = CNCy$**
X = Cl	*cis*-[$PtCl_2(PPh_3)_2$] (**1**)	*cis*-[$PtCl_2(PPh_3)(CNCy)$] (**4**)	*cis*-[$PtCl_2(CNCy)_2$] (**7**)
X = Br	*cis*-[$PtBr_2(PPh_3)_2$] (**2**)	*cis*-[$PtBr_2(PPh_3)(CNCy)$] (**5**)	*cis*-[$PtBr_2(CNCy)_2$] (**8**)
X = I	*trans*-[$PtI_2(PPh_3)_2$] (**3**)	*cis*-[$PtI_2(PPh_3)(CNCy)$] (**6**)	*trans*-[$PtI_2(CNCy)_2$] (**9**)

The complex *cis*-[$PtCl_2(CNCy)_2$] (**7**) is not subject to isomerization and is pure *cis* in both solution and solid state [2]. The complex *cis*-[$PtBr_2(CNCy)_2$] (**8**) was prepared via a treatment of *cis*-[$PtCl_2(CNCy)_2$] (**7**) with excess KBr to in acetone in good yield (90%). In addition, the supplementation of phosphine to the complex *cis*-[$PtBr_2(CNCy)_2$] **8** haven't been resulted to the ligand exchange of isocyanide for phosphine.

The resulting compounds **1–9** are air- and moisture-stable at 20–200 °C and well soluble in common aprotic solvents (e.g. CH_2Cl_2, $CHCl_3$, and MeCN). The prepared complexes are uncolored (**1**, **4**, **7**), yellowish (**2**, **5**, **8**) or yellow (**3**, **6**, **9**) solids. The new

species **4–6,8** were characterized by high-resolution ESI$^+$-MS, IR, ^{1}H, ^{13}C{^{1}H, ^{31}P }, ^{195}Pt{^{1}H} and ^{31}P{^{1}H} NMR spectroscopy. The species give satisfactory microanalyses that are consistent with the proposed formulations. In the HR ESI$^+$-MS, the ions [M + Na]$^+$ with the characteristic isotopic distribution were detected. The *cis* structure of **8** can be confirmed by the presence of two stretching vibration at 2261 and 2233 cm^{-1} due to ν(C≡N) in IR spectra suggesting the *cis*-position of the two isocyanide ligands, since IR spectra of **9** has only one vibration of ν(C≡N) at 2261 cm^{-1} consequence of *trans*-orientation of CNCy ligands [8]. Meanwhile, the mixed-ligand **5** and **6** show only one ν(C≡N) bands at 2227 cm^{-1} for **5** and at 2239 cm^{-1} for **6**. The replacement of Cl ligand with Br have been led to no shift in the vibration of the isocyanide group, as complex **7** has ν(C≡N) at 2227 cm^{-1} and the iodide ligand changes these vibrations slightly. The according to IR spectroscopy data, the value of ν(C≡N) in **4–9** is greater than ν(C≡N) in free cyclohexyl isocyanide with 2128 cm^{-1} [9], that indicates a significant increase in the electrophilicity of the isocyanide carbon atom, and thus, it suggests the coordination to a metal [10-12].

The ^{1}H NMR spectra of **4–9** display the characteristic signal of the C*H* protons from the cyclohexyl rings at *ca*. 4 ppm, while in the ^{13}C NMR spectrum, the corresponding *C*H carbon signal emerges at δ_C ca. 55 ppm (see section S7). The introduction of phosphine ligand in **4**–**6** proceeds to exhibition of two multiplets at 7.40–7.80 ppm corresponding for phosphine's phenyl protons. As excepted, the coordination of isocyanide to platinum shifts the ^{13}C{^{1}H} NMR signal of the terminal carbon atom to downfield, so in particular, δC_{CN} for complexes is 112.1 ppm for **7** [2], 106.60 ppm – **8**, 117.2 ppm – **9**, whereas the δC in CNCy is 154 ppm [9]. Herein, these δC_{CN} have a typical structure composing of three lines with equal intensity resulting of the ^{14}N–C coupling ($J_{C,N}$ = 25 Hz) [5, 13, 14]. Remarkably, this signal shifts in downfield after exchange of one isocyanide ligand with phosphine (δC_{CN} 128.65 ppm – **4**, 129.2 – **5**, 134.1 – **6**). The ^{31}P{^{1}H} NMR spectra of complexes **4**–**6** display only single signal at δ_P 8.38 (**4**), 8.79 (**5**) and 7.59 ppm (**6**) with the three various doublets of spin–spin coupling P with Pt, N and C atoms ($J_{P,Pt}$ *ca.* ~3200–3400 Hz, $J_{P,C}$ ~63–65 Hz, $J_{P,N}$ ~11 Hz), which suggest that these complexes exist in only one form in solution [5, 7, 15-17]. Besides, these strong influence of spin–spin coupling with N and P atoms was also observed in the ^{195}Pt NMR spectra. In particular, the ^{195}Pt NMR

spectrum of complex **7** was shown a signal at –4107 ppm, which has a splitting characteristic for isocyanide complexes on ^{14}N atom ($J_{Pt,N}$ = ~100 Hz). In contrast, complexes **4**–**6** have signals as a doublet of triplets δ_{Pt} 4118 (**4**), 4394 (**5**) and 4985 (**6**) ppm in the ^{195}Pt NMR spectra. The influence of the P atom is stronger N atom, since the $J_{Pt,N}$ coupling exhibits ca. 105 Hz in comparison with an averaged $J_{Pt,P}$ coupling at ~3300 Hz [7, 15]. In addition, the ^{195}Pt signals in **8** and **9** were found that the replacing bromine with iodine shifts δ_{Pt} signals to the upfield at *ca*. ~860 ppm.

The *cis* geometric configuration of **5** and **6** were deduced by the fact that its ^{31}P NMR spectra are practically the same from that of **4** (δ_P ca. 7–9 ppm, $J_{P,Pt}$ *ca.* 3200–3400 Hz). Phosphorus-platinum one-bond spin–spin coupling constants ($^1J_{Pt,P}$) are dominated by the Fermi contact interaction of nuclei with *s*-orbital electrons [18, 19] and are used as an estimate of the bond strength (Table S2).

Table S2. ^{31}P and $^1J_{P,Pt}$ NMR data for $[PtX_2(PPh_3)_2]$ and *cis*-$[PtX_2(PPh_3)(CNCy)]$.

δ_P, $^1J_{P,Pt}$, Hz	*cis*/*trans*-$[PtX_2(PPh_3)_2]$	*cis*-$[PtX_2(PPh_3)(CNCy)]$
X = Cl	δ_P = 14.9 ($^1J_{P,Pt}$ = 3672 Hz)	δ_P = 8.4 ($^1J_{P,Pt}$ = 3416 Hz)
X = Br [3]	δ_P = 14.3 ($^1J_{P,Pt}$ = 3614 Hz)	δ_P = 8.5 ($^1J_{P,Pt}$ = 3358 Hz)
X = I [4]	δ_P = 11.89 ($^1J_{P,Pt}$ = 3455 Hz)	δ_P = 7.6 ($^1J_{P,Pt}$ = 3216 Hz)

S2. X-ray diffraction studies

Table S3 Crystal data and structure refinement for **5**, **6**, **8**.

**Identification code**	**4**	**6**	**7**
CCDC	1811734	1839000	1839001
Temperature/K	248.01(10)	299.12(10)	299.12(10)
Crystal system	monoclinic	monoclinic	triclinic
Space group	$P2_1/c$	$P2_1/c$	P-1
a/Å	12.4465(5)	13.3297(3)	14.1865(4)
b/Å	9.5847(3)	10.8521(2)	14.3572(5)
c/Å	13.8166(6)	17.5272(3)	14.3860(5)
α/°	90.00	90.00	116.720(3)
β/°	96.484(4)	109.023(2)	98.499(3)
γ/°	90.00	90.00	93.588(3)
Volume/Å^3	1637.72(11)	2396.94(9)	2560.73(16)
Z	4	4	2
ρ_{calc}g/cm^3	2.325	2.013	2.128
μ/mm^{-1}	13.437	9.267	7.965
F(000)	1072.0	1384.0	1528.0
Radiation	MoKα (λ = 0.71073)	MoKα (λ = 0.71073)	MoKα (λ =0.71073)
Reflections collected	12587	46753	19054
Independent reflections	3198 [Rint = 0.0515]	5432 [R_{int} = 0.0406]	10049 [R_{int} = 0.0213]
Final R indexes [all data]	R_1 = 0.0417, wR_2 = 0.0810	R_1 = 0.0218, wR_2 = 0.0446	R_1 = 0.0358, wR_2 = 0.0569

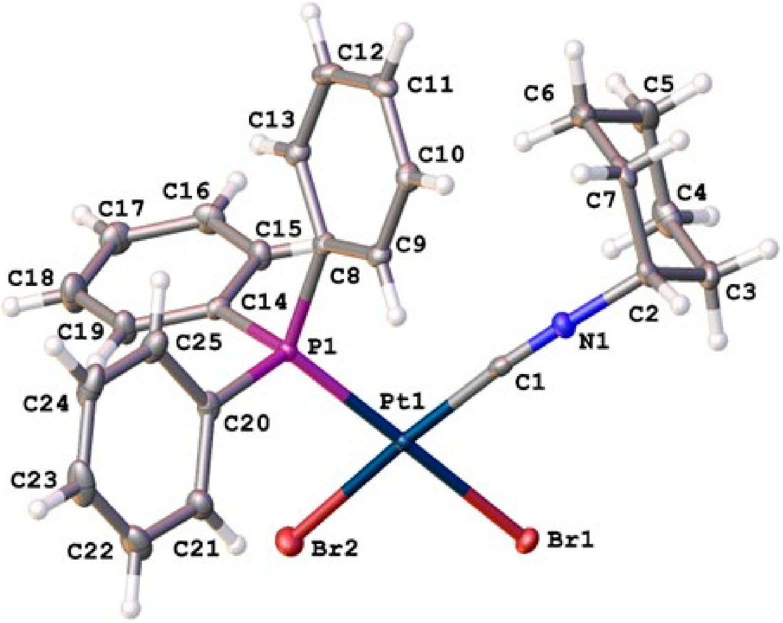

Figure S1. The structures of complex **5**. The thermal ellipsoids are shown at 50% probability.

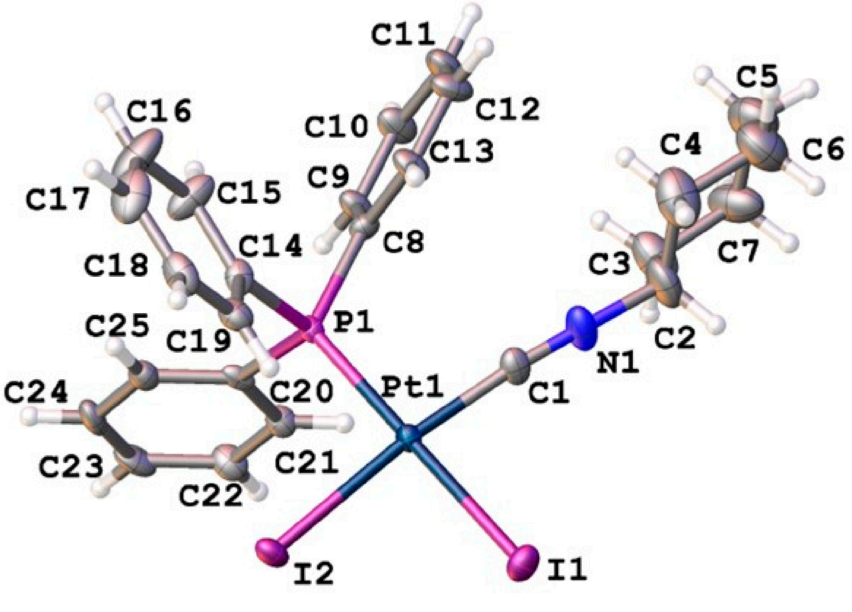

Figure S2. The structures of complex **6**. The thermal ellipsoids are shown at 50% probability.

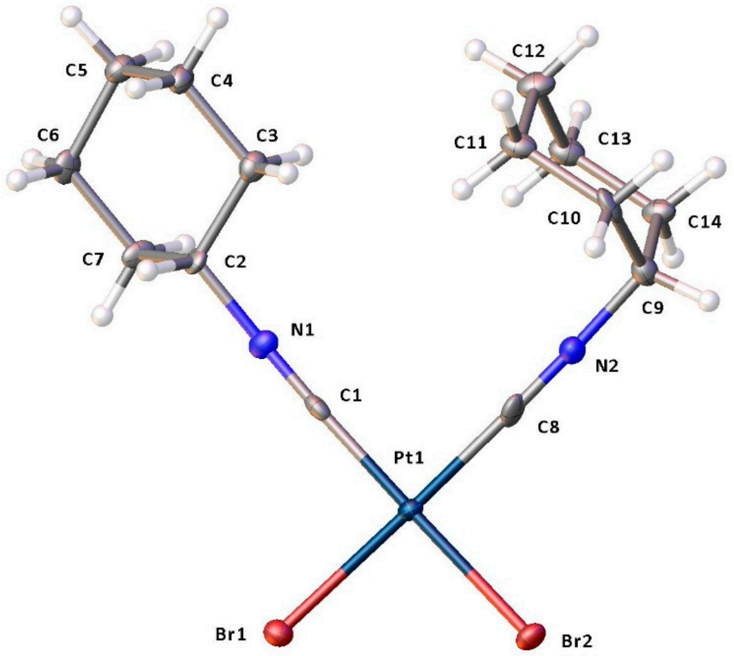

Figure S3. The structures of complex **8**. The thermal ellipsoids are shown at 50% probability.

Table S4. Selected bond lengths (Å) and angles (°) for **5**, **6**, **8**. There are two independent molecules in the unit cell in structure **6**, that are denoted by **A** and **B** letters.

	**5**	**6**		**8**
		**A**	**B**	
Pt1–X1	2.4251(3)	2.6305(4)	2.6251(4)	2.4478(7)
Pt1–X2	2.4884(3)	2.6611(4)	2.6459(4)	2.4541(6)
Pt1–C1	1.918(3)	1.906(5)	1.930(5)	1.916(7)
Pt1–C2				1.920(7)
Pt1–P1	2.2552(7)	2.2634(13)	2.2592(13)	
N1–C1	1.145(4)	1.141(6)	1.144(6)	1.136(8)
N2–C2				1.149(8)
N1–C2	1.460(4)	1.456(6)	1.448(7)	1.466(9)
N2–C9				1.452(8)
∠(X1–Pt1–X2)	89.749(11)	90.526(13)	92.142(12)	91.91(2)
∠(C1–Pt1–X1)	85.71(9)	85.06(15)	85.79(14)	89.27(17)
∠(C2–Pt1–X2)				89.08(19)
∠(P1–Pt1–X2)	88.12(2)	89.78(3)	87.90(3)	
∠(C1–Pt1–C2)				89.8(3)
∠(C1–Pt1–P1)	96.32(9)	94.71(16)	94.24(14)	
∠(Pt1–C1–N1)	173.9(3)	174.5(5)	177.4(4)	178.0(6)
∠(Pt1–C2–N2)				176.5(6)
∠(C1–N1–C3)	173.4(3)	176.4(6)	172.9(5)	174.6(6)
∠(C2–N2–C9)				169.0(6)

S3. CCDC search.

Table S5. Structures of [$PtHal_2$(CNR)(X)] (X = CNR, PR′$_3$) observed by CCDC search.

Structure	R and/or R′	Type of isomer	Ref.
**[$PtCl_2(CNR)_2$]**			
CPICPT	CNPh	*cis*	[20]
CPICPT10			
CPICPT11			
CPICPT12			
KIWQAK	(*o*-$CNC_6H_4O)_2$(*o*-C(O)$_2C_6H_4$-*o*-C(O)$_2$)	*cis*	[16]
OZIPIZ	CNC_6H_4-*p*-Br	*cis*	[21]
PIXMUE	CNtBu, CN[Pt(CNtBu)$_2$Cl]$^+$	*cis*	[22]
QUFVEU	CN-2,6-(2,6-(i-Pr)$_2C_6H_3)_2$-C_6H_3)	*trans*	[23]
QUFVIY	CN-2,6-(2,6-(i-Pr)$_2C_6H_3)_2$-C_6H_3)	*trans*	[23]
REJTAD	CNC_6H_4-*p*-F	*cis*	[24]
REJTAD01			
REJVIN	CNC_6H_4-*p*-OMe	*cis*	
REJVUZ			
VIDGIA	CNCy	*cis*	[2]
VIDGOG	CNtBu	*cis*	
VILYOH	CNXyl	*cis*	[25]
VITZOO			[26]
VITZOO01			[24]
VITZOO02			[27]
**[$PtI_2(CNR)_2$]**			
BOKQUN	CN-2,6-(i-Pr)$_2C_6H_3$-*p*-Br	*trans*	[11]
LAWBAM	CNXyl	*trans*	[28]
LAXQOR			
MAGCIH	CNC_6H_2-2,6-Me_2-4-CCC_6H_4-(4-OCHMe($C_2H_4CH_3$))	*trans*	[29]
MAGCON	CNC_6H_2-2,6-Me_2-4-CCC_6H_4-(4-OCHMe($C_4H_8CH_3$))	*trans*	
MAGCUT	CNC_6H_2-2,6-Me_2-4-CCC_6H_2-(3,5-Me_2-4-OCHMe($C_4H_8CH_3$))	*trans*	
PUBJUR	CNC_6H_2-2,6-iPr_2-4-CNC_6H_4-(4-O($CH_2)_3$)	*trans*	[30]
PUBKAY	CNC_6H_2-2,6-iPr_2-4-CNC_6H_4-(4-O($CH_2)_7$)	*trans*	
TOWXIP	CNC_6H_4-*p*-F	*trans*	[31]
TOWXOV	CNC_6H_4-*p*-Br	*trans*	
TOWXUB	CNC_6H_4-*p*-I	*trans*	
TOWZIR	CNC_6H_4-*p*-F	*trans*	
TOWZUD	CNC_6H_4-*p*-Br	*trans*	
TOXMAX	CNC_6H_4-*p*-Cl	*trans*	
YISBEH	CNC_6H_4-4-C_6H_4-(4-O($CH_2)_5$Et)	*trans*	[32]
**[$PtCl_2$(CNR)(PR′$_3$)]**			
LUJLUX	[Fe(CO)$_4${μ-dppm}], CNCy	*cis*	[15]
LUJMAE	[Fe(CO)$_4${μ-dppm}], $CNCH_2$-*p*-Tos	*cis*	
VILMEL	PPh_3, CNMes	*cis*	[25]
VILQAL	PPh(2-Br-C_2H_2C(Me)$_2$-*o*-C6H4), CNMes	*cis*	
**[PtI_2(CNR)(PR′$_3$)]**			
DUWGUX	PPh_3, 2,2′-CN-1,1′-$C_{10}H_6$	*trans*	[33]

S4. Theoretical studies

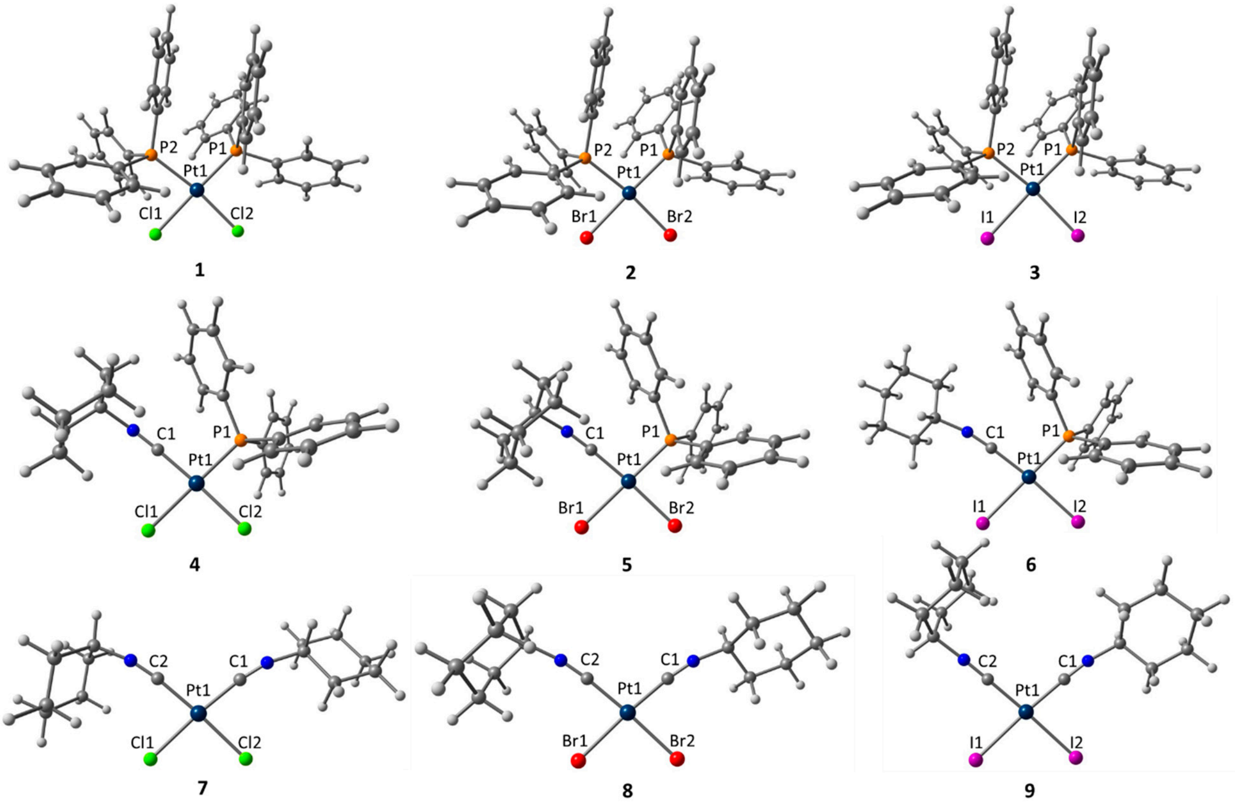

Figure S4. View of optimized structures of complexes **1**–**9**.

Table S6. Bond orders and QTAIM of complexes **1**–**9**, where values of the density of all electrons – ρ(**r**), Laplacian of electron density – ∇²ρ(**r**), energy density – H_b, potential energy density – V(**r**), Lagrangian kinetic energy – G(**r**) (a.u.), MBO – Mayer bond order, and WI – Wiberg bond indices.

Bond	Optimized bond lengths, Å	Experimental bond lengths, Å	MBO	WI	ϱ	G(r)	V(r)	H(r)	∇²ϱ(r))	∣V(r)∣/G(r)
**1**										
Pt1–P1	2.30070	2.26051	0.7300	1.066	0.116	0.071	–0.127	–0.056	0.059	1.79
Pt1–P2	2.32539	2.27121	0.6888	1.026	0.111	0.066	–0.118	–0.052	0.061	1.79
Pt1–Cl1	2.38676	2.35093	0.6800	1.093	0.087	0.071	–0.100	–0.029	0.173	1.41
Pt1–Cl2	2.36146	2.34837	0.7279	1.185	0.092	0.075	–0.106	–0.032	0.176	1.41
**2**										
Pt1–P1	2.31860	2.27752	0.7118	1.031	0.112	0.068	–0.121	–0.053	0.064	1.78
Pt1–P2	2.34296	2.29476	0.6971	0.997	0.107	0.064	–0.112	–0.048	0.066	1.75
Pt1–Br1	2.51929	2.48488	0.6591	1.147	0.078	0.052	–0.076	–0.023	0.118	1.46
Pt1–Br2	2.49660	2.47295	0.6610	1.227	0.082	0.055	–0.080	–0.026	0.118	1.45
**3**										
Pt1–P1	2.33910	2.27694	0.6871	0.998	0.108	0.065	–0.114	–0.048	0.069	1.75
Pt1–P2	2.36601	2.29202	0.6547	0.962	0.102	0.062	–0.105	–0.044	0.072	1.69

Pt1–I1	2.72346	2.65575	0.6680	1.131	0.066	0.033	–0.051	–0.018	0.062	1.55
Pt1–I2	2.69712	2.63425	0.6603	1.211	0.070	0.035	–0.055	–0.020	0.061	1.57
**4**										
Pt1–P1	2.30691	-	0.8866	1.028	0.116	0.068	–0.124	–0.056	0.049	1.82
Pt1–C1	1.90854	-	1.0264	1.428	0.171	0.185	–0.289	–0.105	0.335	1.56
Pt1–Cl1	2.37815	-	0.6986	1.151	0.089	0.071	–0.101	–0.030	0.169	1.42
Pt1–Cl2	2.36564	-	0.6711	1.146	0.091	0.076	–0.107	–0.031	0.181	1.41
**5**										
Pt1–P1	2.31893	2.25513	0.8682	1.004	0.113	0.066	–0.120	–0.053	0.053	1.81
Pt1–C1	1.91919	1.91757	0.9955	1.394	0.168	0.179	–0.279	–0.100	0.327	1.56
Pt1–Br1	2.51270	2.48843	0.6763	1.187	0.080	0.052	–0.077	–0.024	0.114	1.48
Pt1–Br2	2.49730	2.42508	0.6832	1.202	0.081	0.056	–0.082	–0.026	0.121	1.46
**6**										
Pt1–P1	2.33083	2.25904	0.8141	0.983	0.110	0.064	–0.115	–0.051	0.057	1.80
Pt1–C1	1.93687	1.92887	0.9355	1.347	0.161	0.171	–0.264	–0.093	0.322	1.54
Pt1–I1	2.70992	2.66087	0.6811	1.192	0.069	0.035	–0.056	–0.020	0.061	1.60
Pt1–I2	2.69764	2.63031	0.6686	1.166	0.067	0.034	–0.053	–0.019	0.061	1.56
**7**										
Pt1–C1	1.93062	1.91062	0.9703	1.343	0.164	0.172	–0.269	–0.097	0.314	1.56
Pt1–C2	1.93497	1.90393	0.9572	1.335	0.162	0.171	–0.266	–0.095	0.318	1.56
Pt1–Cl1	2.36609	2.32071	0.6820	1.163	0.091	0.074	–0.106	–0.032	0.171	1.43
Pt1–Cl2	2.35909	2.31196	0.7101	1.180	0.093	0.075	–0.108	–0.033	0.171	1.44
**8**										
Pt1–C1	1.94243	1.91544	0.9411	1.310	0.160	0.167	–0.259	–0.092	0.314	1.55
Pt1–C2	1.94332	1.92021	0.9369	1.310	0.159	0.167	–0.259	–0.091	0.316	1.55
Pt1–Br1	2.49404	2.44784	0.6984	1.220	0.083	0.055	–0.082	–0.027	0.113	1.49
Pt1–Br2	2.49453	2.45411	0.7097	1.220	0.083	0.055	–0.081	–0.027	0.113	1.47
**9**										
Pt1–C1	1.95466	-	0.9001	1.282	0.155	0.163	–0.250	–0.087	0.315	1.53
Pt1–C2	1.95362	-	0.9063	1.285	0.156	0.164	–0.251	–0.087	0.316	1.53
Pt1–I1	2.68852	-	0.7198	1.215	0.071	0.035	–0.056	–0.021	0.054	1.60
Pt1–I2	2.69294	-	0.7150	1.202	0.070	0.035	–0.056	–0.021	0.055	1.60

Table S7. Cartesian atomic coordinates for optimized structures.

Complex 1

**Atom**	**X**	**Y**	**Z**
Pt	2.797819000	5.038449000	8.609659000
Cl	3.099470000	7.405999000	8.591203000
Cl	1.249910000	5.297056000	10.374199000
P	4.429744000	5.077850000	6.953548000
P	2.422335000	2.768883000	8.645969000
C	1.199252000	2.086981000	9.830449000
C	1.547956000	1.203409000	10.853592000
H	2.590082000	0.903016000	10.999505000
C	0.563159000	0.691718000	11.699790000
H	0.844619000	0.000222000	12.499131000
C	-0.766994000	1.057886000	11.527124000
H	-1.536773000	0.656547000	12.192606000
C	-1.118321000	1.940926000	10.505175000

H	-2.161879000	2.238238000	10.370066000
C	-0.141862000	2.456623000	9.663691000
H	-0.419452000	3.162516000	8.873992000
C	3.961893000	1.874417000	9.079129000
C	4.428680000	0.708196000	8.470314000
H	3.908381000	0.284694000	7.606819000
C	5.587009000	0.090093000	8.939532000
H	5.950380000	-0.816683000	8.446491000
C	6.281687000	0.624458000	10.020998000
H	7.187202000	0.133007000	10.388559000
C	5.822650000	1.791317000	10.631659000
H	6.363281000	2.218364000	11.481024000
C	4.672103000	2.416062000	10.161197000
H	4.305445000	3.331878000	10.643318000
C	1.741014000	2.134806000	7.065249000
C	1.354074000	3.052932000	6.084504000
H	1.482737000	4.125274000	6.271777000
C	0.814560000	2.614015000	4.876840000
H	0.522899000	3.345504000	4.117193000
C	0.653239000	1.250739000	4.643645000
H	0.238513000	0.901719000	3.693534000
C	0.993452000	0.329779000	5.635406000
H	0.834686000	-0.739394000	5.467986000
C	1.516531000	0.768724000	6.848129000
H	1.735071000	0.042341000	7.638275000
C	3.862835000	5.807086000	5.362697000
C	4.568076000	5.584477000	4.170666000
H	5.469452000	4.962692000	4.170111000
C	4.123105000	6.137562000	2.973114000
H	4.683356000	5.955962000	2.051391000
C	2.966166000	6.914149000	2.950015000
H	2.614062000	7.344581000	2.007954000
C	2.261935000	7.141528000	4.129448000
H	1.356794000	7.755231000	4.120167000
C	2.705742000	6.595441000	5.332068000
H	2.165095000	6.799749000	6.262313000
C	5.879454000	6.059656000	7.500810000
C	6.210599000	6.065095000	8.860219000
H	5.549654000	5.570520000	9.580352000
C	7.360106000	6.714983000	9.297930000
H	7.608417000	6.717259000	10.362911000
C	8.179098000	7.378329000	8.385579000
H	9.076978000	7.898086000	8.732188000
C	7.844236000	7.389678000	7.034197000
H	8.475396000	7.920539000	6.315773000
C	6.699832000	6.729704000	6.590533000
H	6.443522000	6.753081000	5.526939000

C	5.236323000	3.507336000	6.407883000
C	6.468159000	3.114609000	6.945711000
H	6.970037000	3.742340000	7.688835000
C	7.074298000	1.929336000	6.534883000
H	8.037691000	1.641010000	6.965868000
C	6.463907000	1.121062000	5.579584000
H	6.947535000	0.196405000	5.250596000
C	5.235541000	1.499949000	5.041277000
H	4.746356000	0.876867000	4.286250000
C	4.626183000	2.681862000	5.452758000
H	3.671240000	2.969214000	5.003496000

Complex 2

Atom	X	Y	Z
Pt	-12.049976000	0.198992000	-10.753787000
Br	-11.767895000	2.701698000	-10.692983000
Br	-13.548097000	0.391139000	-8.765896000
P	-12.433881000	-2.087234000	-10.794883000
P	-10.470734000	0.278969000	-12.482681000
C	-9.014885000	1.270192000	-11.967591000
C	-8.321918000	2.098824000	-12.851738000
C	-7.191898000	2.791730000	-12.419739000
C	-6.745404000	2.657569000	-11.108468000
C	-7.438613000	1.836071000	-10.220384000
C	-8.573021000	1.152950000	-10.644399000
C	-11.089653000	1.012239000	-14.049681000
C	-10.372774000	0.868282000	-15.246575000
C	-10.862501000	1.410248000	-16.431458000
C	-12.076175000	2.096047000	-16.436443000
C	-12.792897000	2.244935000	-15.252039000
C	-12.302886000	1.709942000	-14.062299000
C	-9.666805000	-1.280383000	-13.070886000
C	-8.442629000	-1.697248000	-12.533715000
C	-7.838237000	-2.872374000	-12.974333000
C	-8.443449000	-3.648684000	-13.958897000
C	-9.663858000	-3.245971000	-14.497622000
C	-10.270872000	-2.072935000	-14.057476000
C	-10.892758000	-2.988574000	-10.378853000
C	-10.190598000	-2.475726000	-9.277067000
C	-9.034485000	-3.103621000	-8.824489000
C	-8.561363000	-4.244709000	-9.471954000
C	-9.247869000	-4.750194000	-10.572248000
C	-10.410940000	-4.128630000	-11.024923000
C	-13.105277000	-2.671334000	-12.399416000
C	-13.311463000	-4.030895000	-12.669735000
C	-13.836533000	-4.428600000	-13.895740000

C	-14.196859000	-3.474371000	-14.848464000
C	-14.054327000	-2.119318000	-14.561828000
C	-13.513852000	-1.722086000	-13.340324000
C	-13.666521000	-2.810535000	-9.645103000
C	-13.313103000	-3.632265000	-8.573751000
C	-14.300733000	-4.141553000	-7.730146000
C	-15.638694000	-3.834633000	-7.952381000
C	-15.994986000	-3.014771000	-9.023729000
C	-15.015261000	-2.502476000	-9.863497000
H	-8.670141000	2.226636000	-13.880876000
H	-6.661945000	3.447846000	-13.116137000
H	-5.860338000	3.205002000	-10.771812000
H	-7.104789000	1.741218000	-9.183408000
H	-9.140168000	0.537620000	-9.936336000
H	-9.426530000	0.316678000	-15.258359000
H	-10.293210000	1.291713000	-17.357863000
H	-12.462895000	2.517501000	-17.368921000
H	-13.741957000	2.788159000	-15.247051000
H	-12.857184000	1.848859000	-13.127670000
H	-7.942498000	-1.095854000	-11.768619000
H	-6.880332000	-3.177948000	-12.543012000
H	-7.961812000	-4.565788000	-14.311100000
H	-10.148232000	-3.842428000	-15.276797000
H	-11.218506000	-1.765972000	-14.508945000
H	-10.566224000	-1.579400000	-8.765692000
H	-8.500613000	-2.698982000	-7.959997000
H	-7.651468000	-4.738416000	-9.118502000
H	-8.873480000	-5.635834000	-11.094659000
H	-10.921645000	-4.527552000	-11.905623000
H	-13.078533000	-4.784889000	-11.910311000
H	-13.981097000	-5.492525000	-14.104437000
H	-14.612503000	-3.791845000	-15.809171000
H	-14.359614000	-1.360518000	-15.288893000
H	-13.401194000	-0.656254000	-13.112456000
H	-12.264743000	-3.882720000	-8.385807000
H	-14.015445000	-4.783597000	-6.891904000
H	-16.410282000	-4.232740000	-7.287125000
H	-17.044676000	-2.763310000	-9.198597000
H	-15.298062000	-1.840917000	-10.689718000

Complex 3

Atom	X	Y	Z
Pt	-4.087803000	-4.412231000	4.878162000
I	-4.576393000	-6.449103000	3.137573000
I	-3.368192000	-6.302565000	6.662336000
P	-3.563630000	-2.731751000	6.418491000

P	-4.996059000	-3.000854000	3.210508000
C	-2.788467000	-3.155565000	8.028198000
C	-1.442667000	-3.543780000	8.022622000
C	-0.815194000	-3.915416000	9.204700000
C	-1.526227000	-3.910932000	10.404646000
C	-2.862367000	-3.524607000	10.415914000
C	-3.494766000	-3.145345000	9.232346000
C	-5.098147000	-1.857494000	6.918286000
C	-5.236997000	-0.474781000	7.054975000
C	-6.442526000	0.068531000	7.496139000
C	-7.516704000	-0.759824000	7.808028000
C	-7.388180000	-2.141417000	7.668530000
C	-6.188522000	-2.687600000	7.221946000
C	-2.333260000	-1.523468000	5.790107000
C	-1.665115000	-1.795663000	4.593504000
C	-0.697085000	-0.919197000	4.105647000
C	-0.385919000	0.236535000	4.816827000
C	-1.014439000	0.493055000	6.036177000
C	-1.968066000	-0.390968000	6.531051000
C	-3.963804000	-2.814957000	1.702462000
C	-4.304946000	-1.883005000	0.711228000
C	-3.503544000	-1.729273000	-0.416199000
C	-2.347116000	-2.494898000	-0.560840000
C	-1.998215000	-3.417377000	0.421613000
C	-2.804205000	-3.580797000	1.546811000
C	-6.621650000	-3.660288000	2.672290000
C	-6.979852000	-3.793715000	1.329801000
C	-8.216340000	-4.338331000	0.987687000
C	-9.103356000	-4.746198000	1.979447000
C	-8.749978000	-4.615650000	3.321688000
C	-7.512457000	-4.084402000	3.666653000
C	-5.399497000	-1.231985000	3.583991000
C	-6.690993000	-0.839330000	3.953905000
C	-6.975491000	0.498106000	4.221884000
C	-5.977773000	1.463982000	4.124734000
C	-4.687995000	1.084292000	3.757509000
C	-4.402015000	-0.250880000	3.487591000
H	-0.884498000	-3.573230000	7.080110000
H	0.233964000	-4.223151000	9.187786000
H	-1.034278000	-4.211571000	11.334127000
H	-3.424634000	-3.517215000	11.353982000
H	-4.546867000	-2.846652000	9.258160000
H	-4.415124000	0.196200000	6.791381000
H	-6.540954000	1.154557000	7.587163000
H	-8.459426000	-0.328957000	8.157536000
H	-8.227036000	-2.800468000	7.909995000
H	-6.086164000	-3.775894000	7.115232000

H	-1.910860000	-2.706654000	4.036237000
H	-0.189144000	-1.145779000	3.163400000
H	0.367024000	0.931183000	4.433359000
H	-0.746255000	1.381725000	6.614712000
H	-2.416323000	-0.207777000	7.513210000
H	-5.201423000	-1.263507000	0.825289000
H	-3.782116000	-1.002530000	-1.184697000
H	-1.715745000	-2.369999000	-1.445385000
H	-1.095478000	-4.024668000	0.312201000
H	-2.540922000	-4.325446000	2.306342000
H	-6.281580000	-3.505838000	0.538673000
H	-8.480976000	-4.454831000	-0.067106000
H	-10.069542000	-5.180151000	1.706707000
H	-9.434006000	-4.950722000	4.106269000
H	-7.219160000	-4.026214000	4.721848000
H	-7.494663000	-1.577833000	4.024406000
H	-7.992841000	0.783476000	4.506073000
H	-6.206165000	2.514634000	4.327269000
H	-3.895661000	1.833472000	3.666125000
H	-3.389947000	-0.523052000	3.174937000

Complex 4

**Atom**	**X**	**Y**	**Z**
Pt	1.066988000	5.095735000	8.816118000
P	0.402992000	6.516466000	10.508001000
N	2.730717000	7.161078000	7.260125000
C	2.139366000	6.384196000	7.903764000
C	3.101998000	8.105026000	6.251041000
H	4.062695000	8.555210000	6.558955000
C	0.962714000	8.242876000	10.222100000
C	1.016095000	6.138528000	12.190117000
C	2.334450000	8.522799000	10.297798000
H	3.040170000	7.724274000	10.556808000
C	-1.412098000	6.662960000	10.631516000
C	1.253983000	4.808897000	12.557699000
H	1.077219000	4.006172000	11.833287000
C	-2.182182000	6.477855000	9.477878000
H	-1.699137000	6.160182000	8.546854000
C	1.913823000	10.826295000	9.712967000
H	2.284026000	11.837512000	9.520923000
C	2.020973000	9.185043000	6.169334000
H	1.909803000	9.674052000	7.153084000
H	2.388431000	9.955068000	5.465281000
C	2.806311000	9.806896000	10.049911000
H	3.877577000	10.015768000	10.123092000
C	0.074622000	9.266312000	9.879726000

H	-0.999812000	9.064818000	9.816811000
C	3.264759000	7.376557000	4.915973000
H	4.019230000	6.579097000	5.019076000
H	3.669094000	8.114155000	4.197411000
C	1.219021000	7.163518000	13.125505000
H	1.039248000	8.208101000	12.848721000
C	-3.558692000	6.677161000	9.521039000
H	-4.156279000	6.526495000	8.617863000
C	-2.032579000	7.031296000	11.828670000
H	-1.440186000	7.163282000	12.739987000
C	1.940762000	6.818634000	4.412854000
H	2.090169000	6.355718000	3.423603000
H	1.611600000	5.996044000	5.077162000
C	0.551995000	10.553011000	9.624685000
H	-0.151995000	11.347969000	9.362533000
C	0.873462000	7.903720000	4.338755000
H	-0.085493000	7.476538000	4.001795000
H	1.166072000	8.649288000	3.572098000
C	0.699024000	8.617184000	5.673683000
H	-0.046066000	9.426567000	5.593346000
H	0.295499000	7.909473000	6.425338000
C	1.653853000	6.860158000	14.412392000
H	1.810150000	7.665146000	15.136171000
C	1.892475000	5.535167000	14.773507000
H	2.238958000	5.298192000	15.783646000
C	1.691439000	4.514880000	13.847399000
H	1.877320000	3.474307000	14.127300000
C	-4.174212000	7.049597000	10.714971000
H	-5.257530000	7.196880000	10.748734000
C	-3.411821000	7.222482000	11.867991000
H	-3.893625000	7.503817000	12.808616000
Cl	1.647641000	3.650989000	7.018576000
Cl	-0.335178000	3.465802000	9.802788000

Complex 5

Atom	X	Y	Z
Pt	1.195282000	5.008854000	8.896026000
Br	1.911633000	3.455811000	7.055216000
Br	-0.238221000	3.265216000	9.964344000
P	0.464068000	6.474024000	10.537984000
N	2.773739000	7.085717000	7.250382000
C	2.222980000	6.311171000	7.931089000
C	3.145889000	7.977546000	6.195918000
H	4.177116000	8.315165000	6.401971000
C	1.013014000	8.200939000	10.224081000
C	1.051079000	6.177435000	12.245505000

C	2.379378000	8.491702000	10.342752000
H	3.081242000	7.701485000	10.635645000
C	-1.356514000	6.596639000	10.600714000
C	1.492278000	4.908802000	12.635604000
H	1.479283000	4.082455000	11.917292000
C	-2.060858000	6.506539000	9.393869000
H	-1.520718000	6.294327000	8.463354000
C	1.961998000	10.786700000	9.723197000
H	2.331101000	11.799047000	9.534859000
C	2.195429000	9.176178000	6.199563000
H	2.226134000	9.670859000	7.185774000
H	2.587389000	9.900385000	5.461160000
C	2.849905000	9.776940000	10.099354000
H	3.916464000	9.994540000	10.206896000
C	0.129289000	9.214508000	9.844204000
H	-0.941570000	9.005724000	9.751796000
C	3.096499000	7.224704000	4.864591000
H	3.745839000	6.335004000	4.920476000
H	3.529182000	7.897616000	4.100537000
C	1.058473000	7.228951000	13.174668000
H	0.727941000	8.230287000	12.876449000
C	-3.442712000	6.662279000	9.378605000
H	-3.986648000	6.586943000	8.433114000
C	-2.050947000	6.819247000	11.792205000
H	-1.514109000	6.867425000	12.744698000
C	1.674405000	6.839295000	4.483058000
H	1.678754000	6.353450000	3.493640000
H	1.301338000	6.070362000	5.186913000
C	0.606019000	10.502265000	9.591837000
H	-0.094831000	11.289778000	9.300096000
C	0.748560000	8.048932000	4.484900000
H	-0.280729000	7.746115000	4.230413000
H	1.067893000	8.754365000	3.691593000
C	0.777271000	8.771185000	5.825543000
H	0.128530000	9.663385000	5.809010000
H	0.362185000	8.111382000	6.614009000
C	1.491410000	7.008564000	14.478136000
H	1.492554000	7.832998000	15.196834000
C	1.928297000	5.741188000	14.862394000
H	2.273673000	5.569777000	15.886068000
C	1.929179000	4.696935000	13.942030000
H	2.274109000	3.702661000	14.238604000
C	-4.132196000	6.892202000	10.568619000
H	-5.220195000	7.003719000	10.557528000
C	-3.437028000	6.964949000	11.772714000
H	-3.976666000	7.130549000	12.709405000

Complex 6

Atom	X	Y	Z
Pt	-3.027643000	9.083505000	7.410427000
I	-4.681897000	9.550625000	9.489495000
I	-4.529794000	10.607112000	5.747353000
P	-1.739870000	7.764918000	8.837216000
N	-1.212982000	8.888299000	4.899396000
C	-1.881329000	8.913725000	5.858455000
C	-0.178863000	7.197270000	8.046553000
C	-1.664732000	9.253026000	2.555424000
H	-2.236359000	10.157263000	2.823334000
H	-2.384574000	8.412254000	2.577198000
C	-0.325717000	10.706043000	11.280944000
H	0.055574000	11.721708000	11.144664000
C	1.079574000	7.687672000	8.402191000
H	1.177898000	8.420119000	9.209356000
C	-0.205457000	8.123835000	0.849492000
H	-0.882128000	7.249560000	0.783351000
H	0.267690000	8.223608000	-0.141593000
C	-2.471474000	6.175988000	9.373775000
C	-0.953308000	8.882024000	12.729487000
H	-1.064421000	8.462523000	13.733280000
C	-0.279238000	6.249013000	7.018589000
H	-1.263219000	5.858923000	6.730706000
C	0.861149000	5.792767000	6.368213000
H	0.771284000	5.043080000	5.576186000
C	2.116174000	6.289388000	6.724650000
H	3.014098000	5.929963000	6.213619000
C	2.221290000	7.237243000	7.737717000
H	3.201942000	7.626507000	8.025991000
C	-0.717582000	9.961681000	10.174221000
H	-0.662810000	10.402895000	9.171695000
C	-1.337217000	8.127535000	11.622206000
H	-1.756103000	7.127266000	11.766999000
C	-0.443623000	10.166115000	12.561321000
H	-0.148902000	10.756860000	13.433278000
C	-1.211522000	8.660700000	10.337569000
C	-3.821227000	5.892215000	9.149871000
H	-4.464232000	6.640988000	8.675906000
C	-1.023694000	9.364255000	1.181025000
H	-0.364795000	10.254373000	1.156381000
H	-1.800553000	9.539301000	0.418950000
C	-4.349042000	4.662756000	9.542279000
H	-5.406143000	4.448940000	9.362779000
C	-1.653799000	5.212545000	9.983735000
H	-0.589227000	5.417604000	10.145316000

C	-3.537158000	3.715792000	10.159001000
H	-3.955326000	2.752997000	10.466774000
C	-2.187422000	3.991228000	10.380297000
H	-1.545024000	3.247039000	10.859532000
C	-0.600299000	8.987926000	3.617685000
H	0.086351000	9.858189000	3.659419000
C	0.846358000	7.860752000	1.918482000
H	1.577210000	8.693235000	1.923982000
H	1.423457000	6.949910000	1.688282000
C	0.217766000	7.741825000	3.298751000
H	-0.461456000	6.867416000	3.335576000
H	0.978802000	7.586492000	4.082558000

Complex 7

Atom	X	Y	Z
Pt	0.308772000	-1.182697000	6.792511000
Cl	-0.861753000	-1.868187000	8.722607000
Cl	-1.270842000	0.505353000	6.288845000
N	1.619017000	-0.065034000	4.218661000
N	2.138037000	-3.484154000	7.747937000
C	1.189306000	-0.540802000	5.193526000
C	1.901546000	0.684350000	3.029676000
H	2.853822000	0.295936000	2.627220000
C	2.048933000	2.165287000	3.384485000
H	2.839014000	2.287536000	4.144414000
H	2.406041000	2.673454000	2.468941000
C	0.731180000	2.773447000	3.842799000
H	0.872823000	3.847578000	4.046983000
H	0.415616000	2.315649000	4.799520000
C	-0.365488000	2.558316000	2.807968000
H	-1.317688000	2.977442000	3.172684000
H	-0.112103000	3.112312000	1.881346000
C	-0.534468000	1.079925000	2.483037000
H	-0.897541000	0.553809000	3.386338000
H	-1.306495000	0.933466000	1.709723000
C	0.777245000	0.466143000	2.015387000
H	1.106649000	0.942575000	1.072851000
H	0.670475000	-0.611635000	1.806383000
C	1.509557000	-2.600961000	7.315892000
C	2.639104000	-4.584274000	8.506225000
H	3.743904000	-4.498421000	8.528429000
C	2.093689000	-4.494689000	9.930644000
H	0.989218000	-4.471811000	9.865735000
H	2.393141000	-3.534104000	10.381391000
C	2.568148000	-5.678097000	10.759073000
H	3.668791000	-5.626149000	10.874707000

H	2.150570000	-5.610739000	11.776864000
C	2.194433000	-7.000039000	10.103744000
H	1.091485000	-7.091219000	10.073430000
H	2.559276000	-7.848676000	10.705800000
C	2.740720000	-7.078049000	8.684938000
H	3.848093000	-7.073608000	8.717567000
H	2.452997000	-8.026636000	8.202660000
C	2.259137000	-5.905648000	7.843689000
H	2.669577000	-5.944310000	6.820841000
H	1.156938000	-5.932436000	7.749561000

Complex 8

Atom	X	Y	Z
Pt	9.737491000	5.214666000	6.136253000
Br	10.810741000	2.964593000	6.061756000
Br	10.419011000	5.765879000	3.800795000
N	8.504985000	8.055916000	5.910045000
C	8.942127000	6.985482000	6.068121000
C	9.262895000	4.694225000	7.947440000
C	8.262108000	9.399025000	3.924665000
H	7.657665000	8.600020000	3.464438000
H	9.315856000	9.165191000	3.679959000
N	9.034691000	4.293446000	9.019192000
C	7.877161000	10.773898000	3.401401000
H	8.006707000	10.805236000	2.307319000
H	6.798970000	10.947958000	3.587707000
C	9.572541000	4.483517000	11.377556000
H	9.122542000	5.490555000	11.389859000
H	9.317398000	4.009678000	12.343543000
C	8.112826000	9.345608000	5.443852000
H	7.045551000	9.481233000	5.711568000
C	11.083959000	4.544333000	11.214708000
H	11.523035000	5.135451000	12.035464000
H	11.332730000	5.085267000	10.281478000
C	8.932473000	10.437344000	6.125859000
H	10.001300000	10.240238000	5.917777000
H	8.802146000	10.376858000	7.219313000
C	8.915813000	3.637643000	10.286955000
H	7.833521000	3.545856000	10.488920000
C	11.061144000	2.314539000	10.055075000
H	11.317440000	2.741915000	9.066415000
H	11.474623000	1.292786000	10.051096000
C	9.547726000	2.246763000	10.196008000
H	9.272708000	1.713519000	11.125501000
H	9.095056000	1.691541000	9.357377000
C	8.687192000	11.871400000	4.077068000

H	9.755267000	11.750004000	3.811779000
H	8.385902000	12.863472000	3.701812000
C	11.686072000	3.146480000	11.166286000
H	12.779478000	3.202603000	11.037149000
H	11.514732000	2.646789000	12.140910000
C	8.538544000	11.805903000	5.590717000
H	7.485722000	12.014993000	5.864954000
H	9.142743000	12.586859000	6.080907000

Complex 9

Atom	X	Y	Z
Pt	7.656328000	4.403620000	5.856145000
N	8.200198000	7.474285000	5.997048000
C	8.010620000	6.322120000	5.976676000
C	8.484826000	3.847180000	7.535611000
C	8.224497000	9.221618000	4.327770000
H	7.240997000	8.876737000	3.968064000
H	8.982048000	8.646801000	3.761245000
N	8.983201000	3.498403000	8.532223000
C	8.421053000	10.714298000	4.112227000
H	8.324336000	10.952206000	3.040434000
H	7.608375000	11.267169000	4.623023000
C	9.009585000	3.750502000	10.944641000
H	7.912822000	3.639151000	10.953044000
H	9.389113000	3.231209000	11.843894000
C	8.379843000	8.874650000	5.806609000
H	7.577612000	9.381848000	6.380344000
C	9.424969000	5.213811000	10.979235000
H	9.017894000	5.696115000	11.883171000
H	8.969971000	5.744500000	10.120545000
C	9.730695000	9.328167000	6.351530000
H	10.520820000	8.761671000	5.822431000
H	9.810152000	9.067127000	7.420406000
C	9.591830000	3.019834000	9.734629000
H	9.342779000	1.945096000	9.791835000
C	11.515153000	4.660023000	9.697244000
H	11.155676000	5.165252000	8.779983000
H	12.614259000	4.743313000	9.677379000
C	11.108910000	3.194413000	9.657650000
H	11.535585000	2.662756000	10.528117000
H	11.494769000	2.689775000	8.756734000
C	9.765084000	11.180883000	4.654342000
H	10.577275000	10.697668000	4.077157000
H	9.886645000	12.266991000	4.508160000
C	10.939884000	5.358562000	10.922068000
H	11.228239000	6.422992000	10.932552000

H	11.381038000	4.911434000	11.834796000
C	9.916685000	10.821029000	6.125828000
H	9.161608000	11.374689000	6.717795000
H	10.899607000	11.139094000	6.510540000
I	7.105493000	1.789438000	5.554866000
I	6.508978000	5.071480000	3.513182000

S5. Catalytic investigations.

Table S8. Photocatalytic activity of complexes observed in this work in the model hydrosilylation reaction.

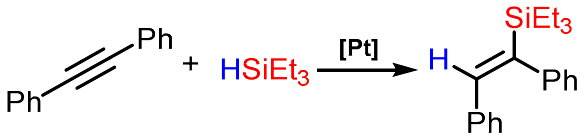

Entry	Catalyst	mol%	τ, h	Product yield[b]	TON / TOF
			**80 °C**		
S1	**1**	0.5	24	99[d]	2×10^2 / 8.3
S2	**2**	0.5	24	99[d]	2×10^2 / 8.3
S3	**3**	0.5	24	97[d]	2×10^2 / 8.3
S4	**4**	0.5	24	99[d]	2.0×10^2 / 8.3
S5	**5**	0.5	24	99[d]	2.0×10^2 / 8.3
S6	**6**	0.5	24	84[d]	1.7×10^2 / 7.1
S7	**7**	0.5	24	21[d]	0.4×10^2 / 1.7
S8	**8**	0.5	24	44[d]	0.9×10^2 / 3.8
S9	**9**	0.5	24	13[d]	0.3×10^2 / 1.3
S10	**1**	0.05	24	98[d]	2.0×10^3 / 81.7
S11	**2**	0.05	24	97[d]	1.9×10^3 / 80.8
S12	**3**	0.05	24	97[d]	1.9×10^3/ 80.8
S13	**4**	0.05	24	99[d]	2.0×10^3 / 82.5
S14	**5**	0.05	24	99[d]	2.0×10^3 / 82.5
S15	**6**	0.05	24	99[d]	2.0×10^3 / 82.5
			**40 °C**		
S16	**1**	0.05	6	20	4.0×10^2 / 66.7
S17	**2**	0.05	6	20	4.0×10^2 / 66.7
S18	**3**	0.05	6	5	1.0×10^2 / 16.7
S19	**4**	0.05	6	98	2.0×10^3 / 326.7
S20	**5**	0.05	6	≤5	–
S21	**6**	0.05	6	≤5	–
S22	**1**	0.5	24	52	1.0×10^2 / 4.3
S23	**2**	0.5	24	96	1.9×10^2 / 8.0
S24	**4**	0.5	24	98	2.0×10^2 / 8.3
S25	**5**	0.5	24	98	2.0×10^2 / 8.3
			**LED 400 nm**		
S26	**1**	0.5	12	57	1.2×10^2 / 4.3
S27	**2**	0.5	12	85	1.7×10^2 / 14.2

S28	**3**	0.5	12	70	$1.4{\times}10^2$ / 11.7
S29	**4**	0.5	12	30	$0.6{\times}10^2$ / 5.0
S30	**5**	0.5	12	98	$2.0{\times}10^2$ / 16.3
S30	**6**	0.5	12	99	$2.0{\times}10^2$ / 16.5
S31	**7**	0.5	12	33	$0.7{\times}10^2$ / 5.5
S32	**8**	0.5	12	10	$0.2{\times}10^2$ / 1.7
S33	**9**	0.5	12	≤5	–
S34	**5**	0.05	24	99	$2.0{\times}10^3$ / 82.5
S35	**6**	0.05	24	99	$2.0{\times}10^3$ / 82.5
S36	**5**	0.05	12	73	$1.5{\times}10^3$ / 121.7
S37	**6**	0.05	12	52	$1.0{\times}10^3$ / 86.7
S38	**1**	0.05	6	38	$7.6{\times}10^2$ / 126.7
S39	**2**	0.05	6	20	$4.0{\times}10^2$ / 66.7
S40	**3**	0.05	6	6	$1.2{\times}10^2$ / 20.0
S41	**4**	0.05	6	15	$3.0{\times}10^2$ / 50.0
S42	**5**	0.05	6	30	$6.0{\times}10^2$ / 100.0
S43	**6**	0.05	6	43	$8.6{\times}10^2$ / 143.3
**LED 450 nm**					
S44	**1**	0.5	12	83	$1.7{\times}10^2$ / 13.8
S45	**2**	0.5	12	58	$1.2{\times}10^2$ / 9.7
S46	**3**	0.5	12	8	$0.2{\times}10^2$ / 1.3
S47	**4**	0.5	12	11	$0.2{\times}10^2$ / 1.8
S48	**5**	0.5	12	14	$0.3{\times}10^2$ / 2.3
S49	**6**	0.5	12	12	$0.2{\times}10^2$ / 2.0
S50	**7**	0.5	12	22	$0.4{\times}10^2$ / 3.6
S51	**8**	0.5	12	20	$0.4{\times}10^2$ / 3.3
S52	**9**	0.5	12	≤5	–
S53	**1**	0.05	12	68	$1.4{\times}10^3$ / 133.3
S54	**1**	0.05	24	99	$2.0{\times}10^3$ / 82.5
S55	**2**	0.05	24	99	$2.0{\times}10^3$/ 82.5
S56	**1**	0.05	6	31	$6.2{\times}10^2$ / 103.3
S57	**2**	0.05	6	23	$4.6{\times}10^2$ / 76.7
S58	**3**	0.05	6	8	$1.6{\times}10^2$ / 26.7
S59	**4**	0.05	6	15	$3.0{\times}10^2$ / 50.0
S60	**5**	0.05	6	8	$1.6{\times}10^2$ / 26.7
S61	**6**	0.05	6	6	$1.2{\times}10^2$ / 20.0

[a]Reaction conditions: 1,2-diphenylacetylene ($5.0{\cdot}10^{-4}$ mol), Et_3SiH ($7.5{\cdot}10^{-4}$ mol); toluene (0.5 mL). [b]Yield of 1,2-(diphenylvinyl)triethylsilane was determined by ^1H NMR spectroscopy using 1,2-dimethoxyethane as the internal standard; product obtained as a mixture of *E*/*Z* isomers; (*E* 75–85%). [d]*E*/*Z* isomers; (*Z* ≤ 5%).

S6 UV/vis absorption spectra

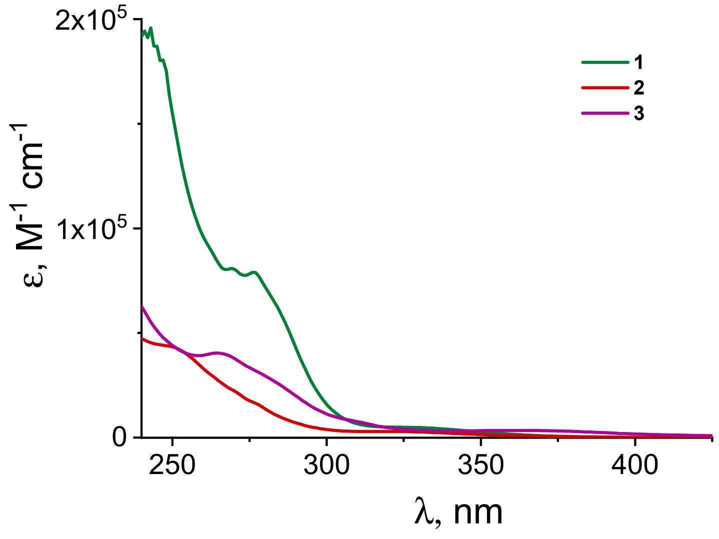

Figure S5. UV/vis absorption spectra of complexes **1–3** in CH_2Cl_2 at RT (2×10^{-5} M).

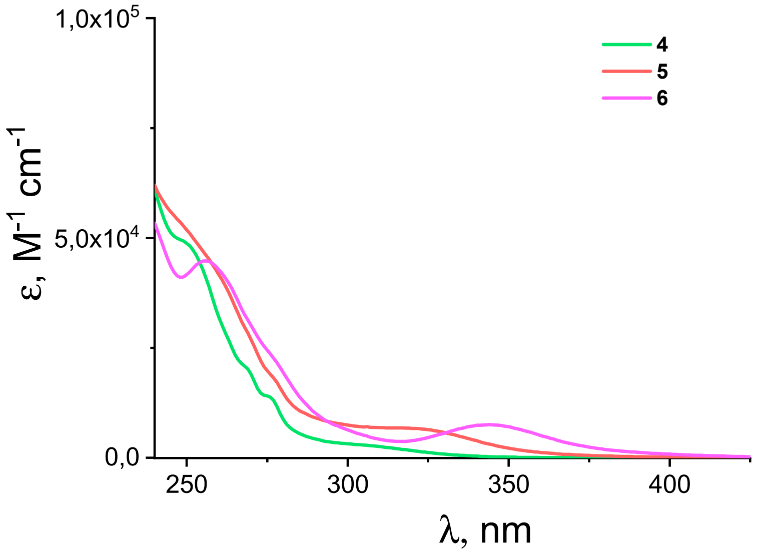

Figure S6. UV/vis absorption spectra of complexes **4–6** in CH_2Cl_2 at RT (2×10^{-5} M).

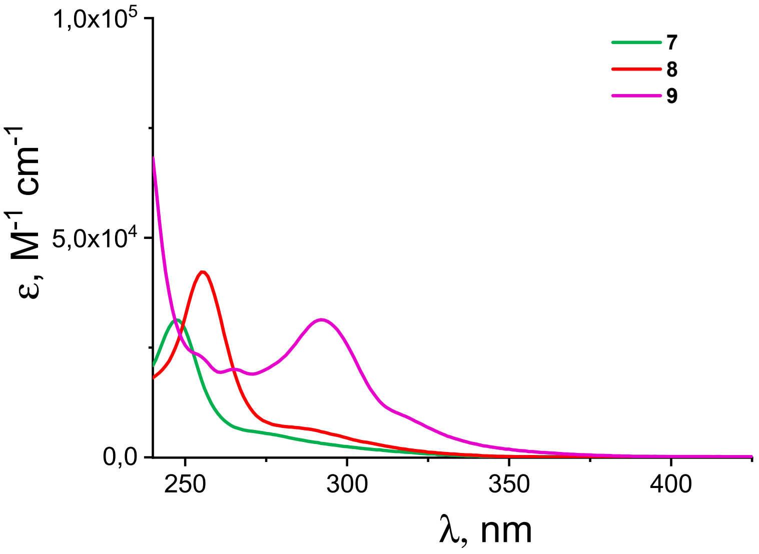

Figure S7. UV/vis absorption spectra of complexes **7–9** in CH_2Cl_2 at RT ($2×10^{-5}$ M).

Table S9. UV-Vis absorption data for **1–9**.

Complex	λ_{max}, nm ($\varepsilon \times 10^{-6}$ M^{-1} cm^{-1})
**1**	241 (1.94), 269 (0.81), 277 (0.79), 327sh (0.05)
**2**	250 (0.43), 323 (0.03)
**3**	265 (0.40), 362 (0.03)
**4**	254 (0.23), 265 (0.20), 293 (0.31), 317sh (0.17)
**5**	247sh (0.55), 321 (0.07)
**6**	256 (0.45), 343 (0.08)
**7**	248 (0.31)
**8**	250sh (0.49), 269 (0.20), 276 (0.14), 306sh (0.03)
**9**	255 (0.42), 284 (0.07)

S7. NMR spectra

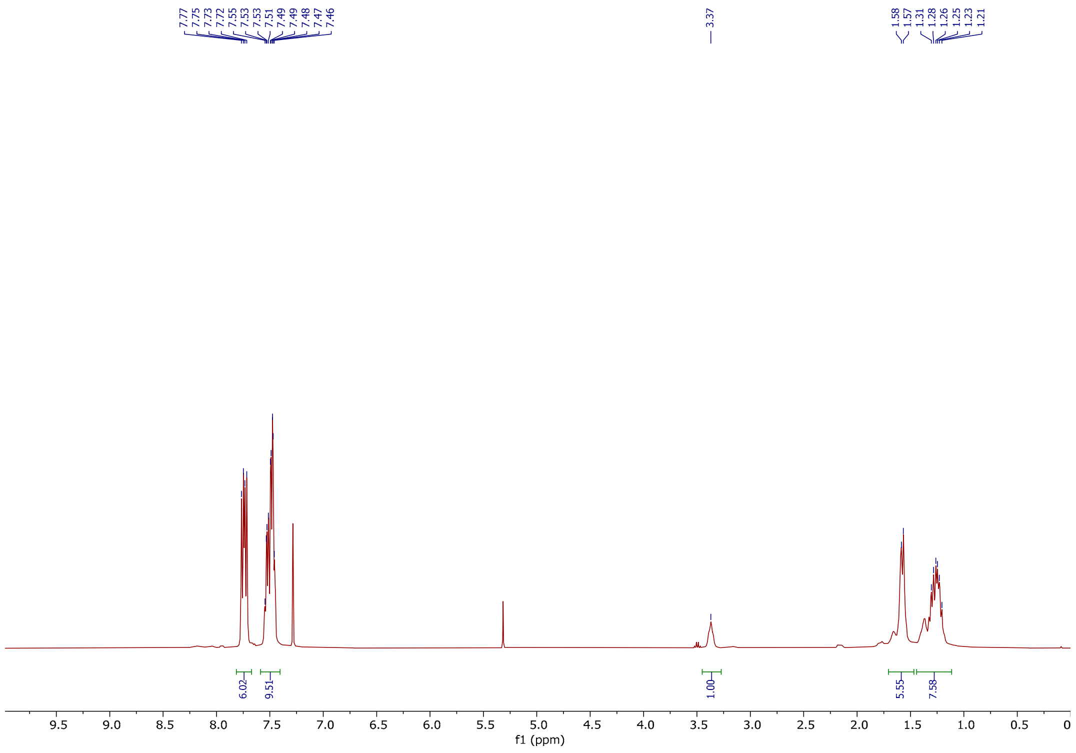

Figure S8. The ^{1}H NMR spectra of $[PtCl_2(CNCy)(PPh_3)_2]$ **4**.

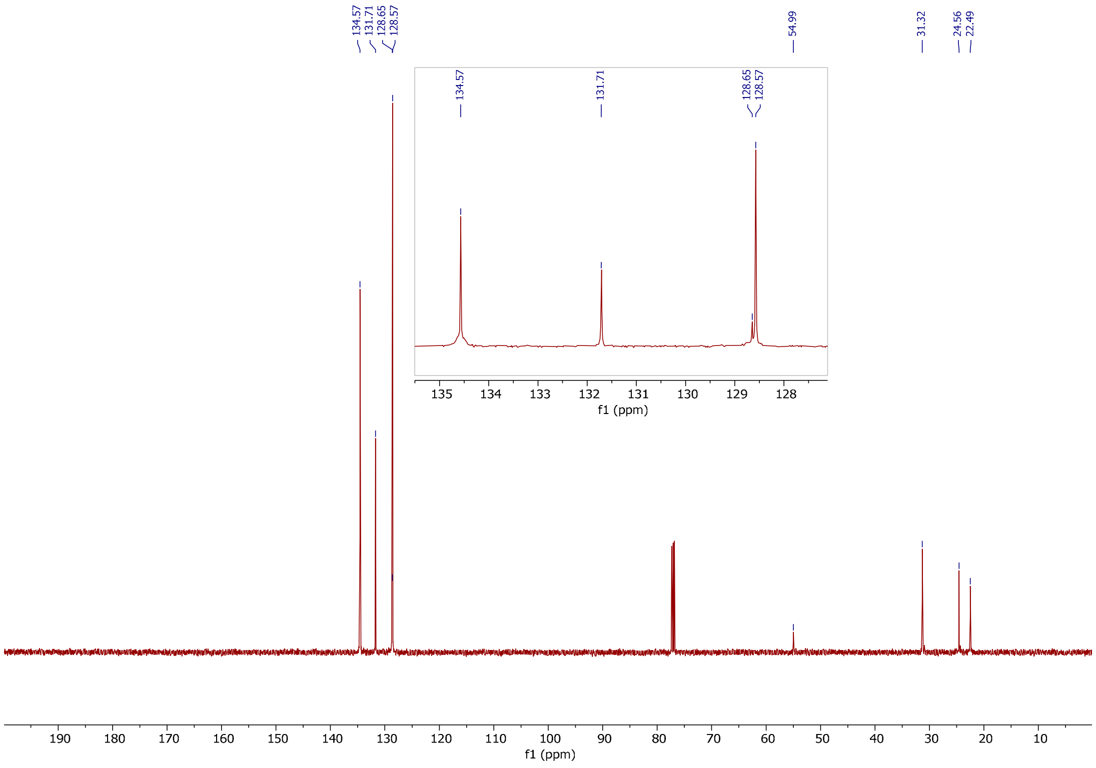

Figure S9. The $^{13}C\{^{1}H, ^{31}P\}$ NMR spectra of $[PtCl_2(CNCy)(PPh_3)_2]$ **4**.

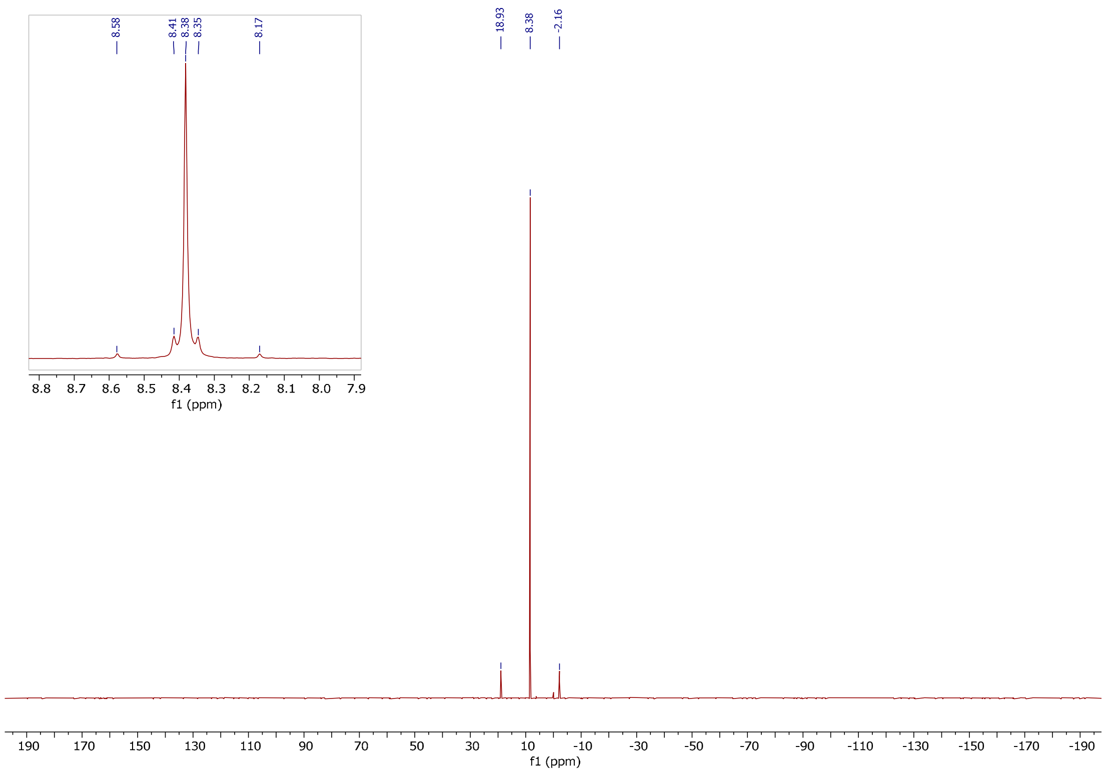

Figure S10. The $^{31}P\{^{1}H\}$ NMR spectra of $[PtCl_2(CNCy)(PPh_3)_2]$ **4**.

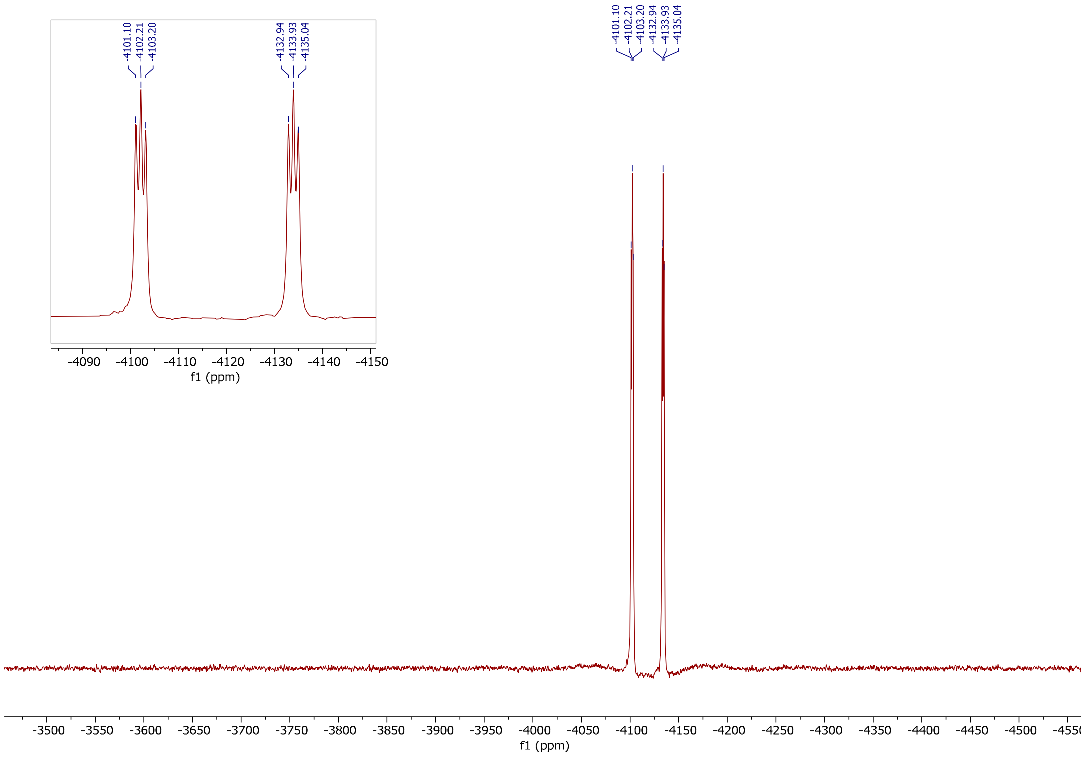

Figure S11. The $^{195}Pt\{^{1}H\}$ NMR spectra of $[PtCl_2(CNCy)(PPh_3)_2]$ **4**.

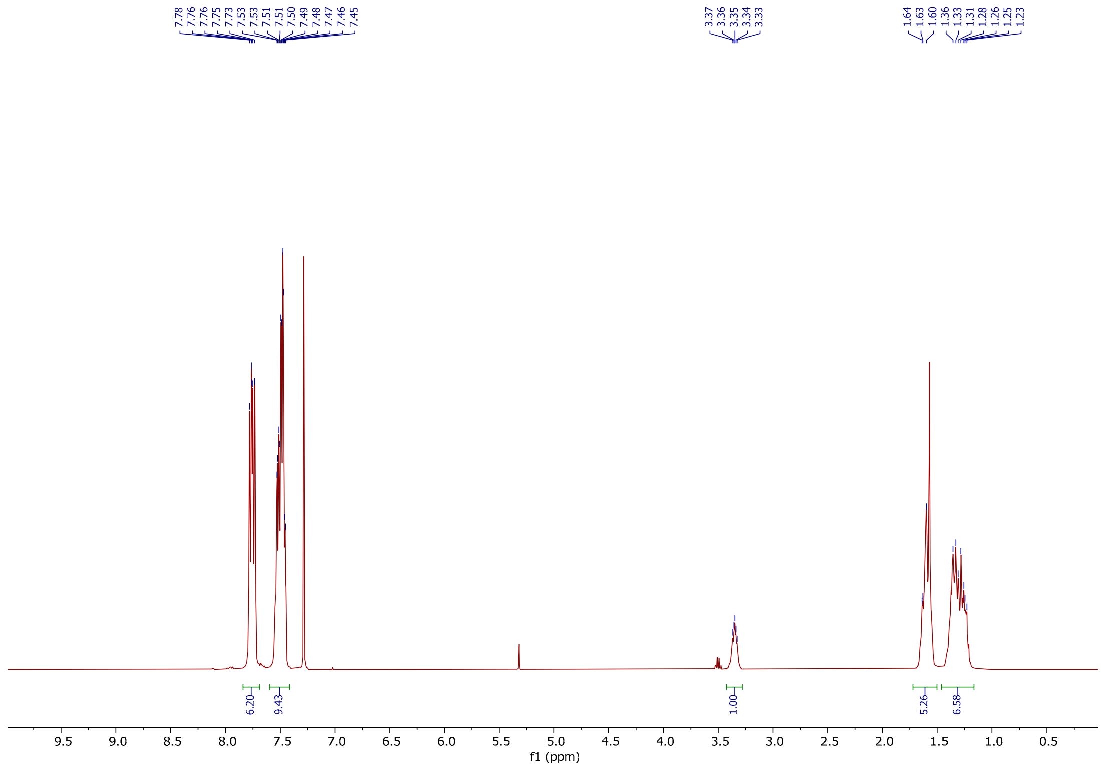

Figure S12. The ^{1}H NMR spectra of $[PtBr_2(CNCy)(PPh_3)_2]$ **5**.

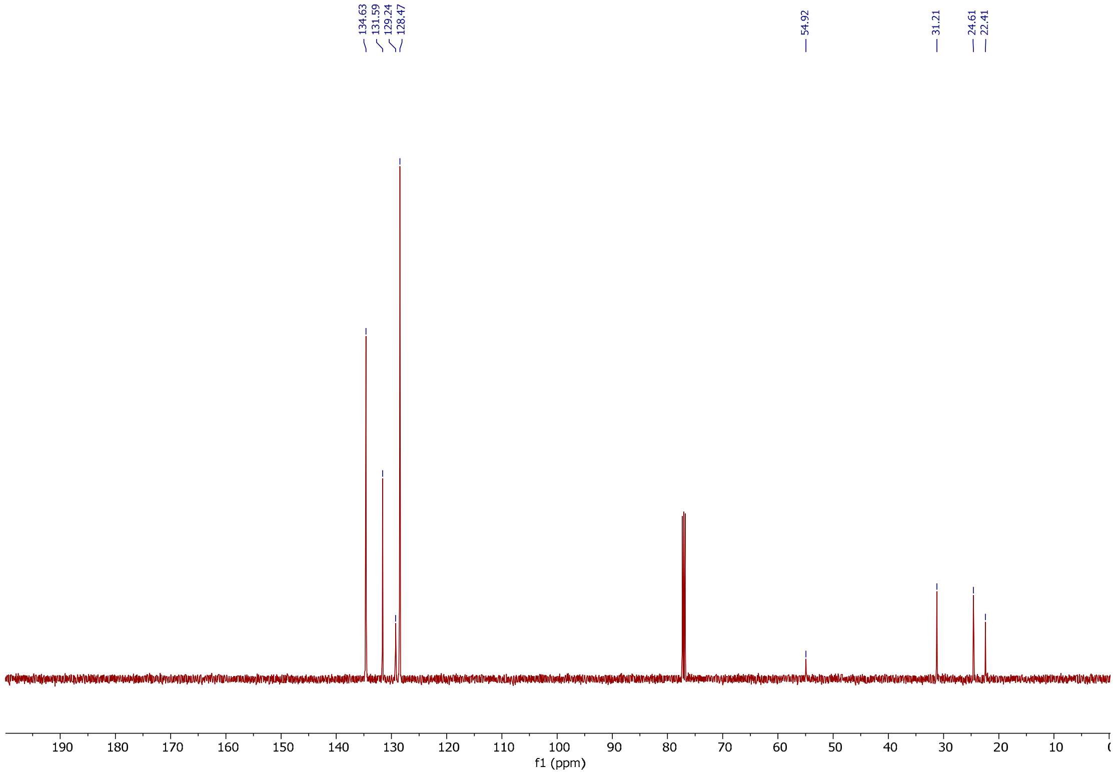

Figure S13. The $^{13}C\{^{1}H, ^{31}P\}$ NMR spectra of $[PtBr_2(CNCy)(PPh_3)_2]$ **5**.

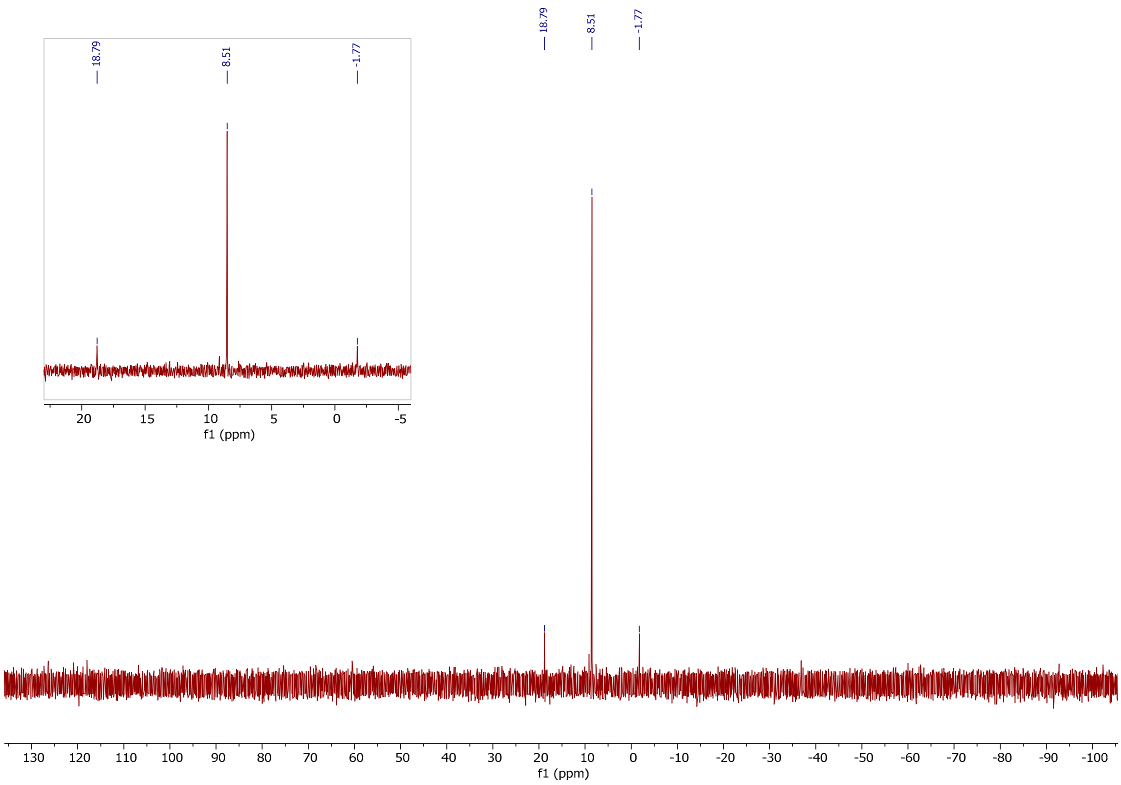

Figure S14. The $^{31}P\{^{1}H\}$ NMR spectra of $[PtBr_2(CNCy)(PPh_3)_2]$ **5**.

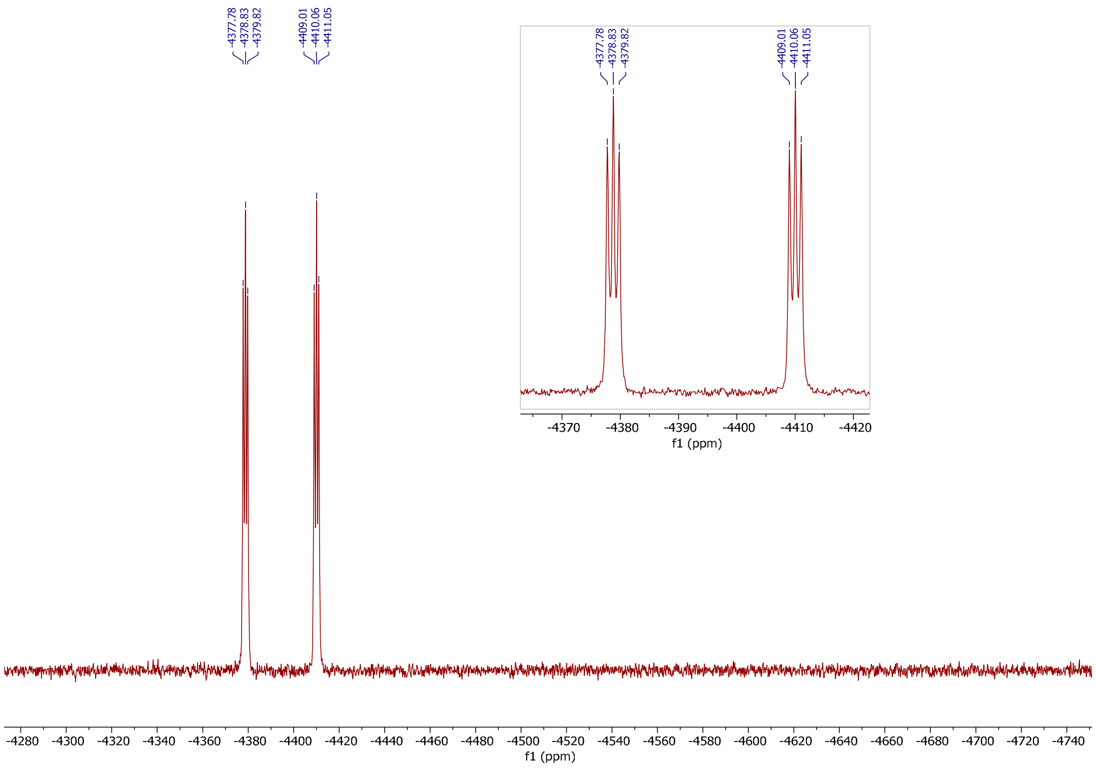

Figure S15. The $^{195}Pt\{^{1}H\}$ NMR spectra of $[PtBr_2(CNCy)(PPh_3)_2]$ **5**.

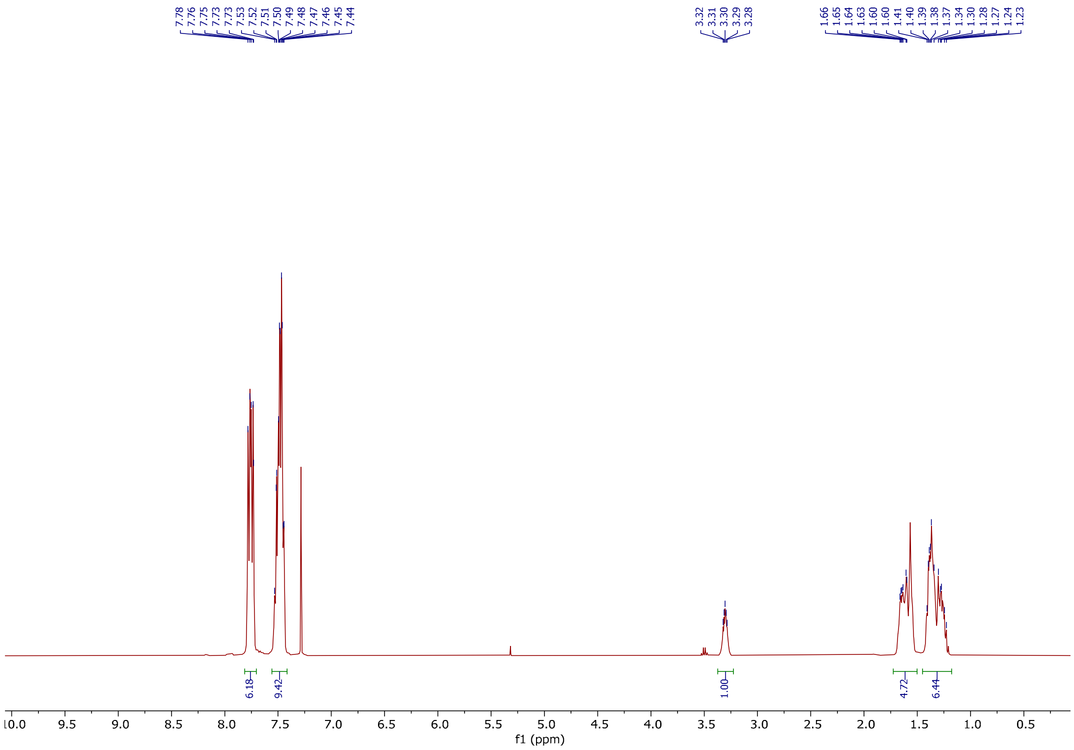

Figure S16. The ^{1}H NMR spectra of $[PtI_2(CNCy)(PPh_3)_2]$ **6**.

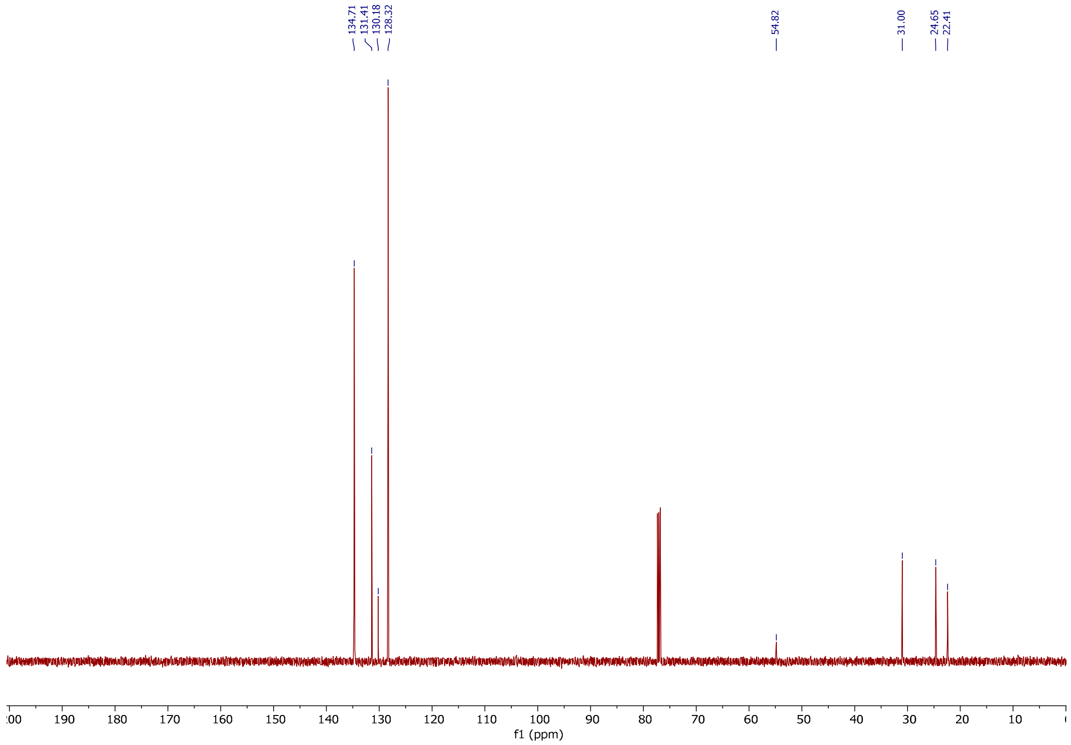

Figure S17. The $^{13}C\{^{1}H, ^{31}P\}$ NMR spectra of $[PtI_2(CNCy)(PPh_3)_2]$ **6**.

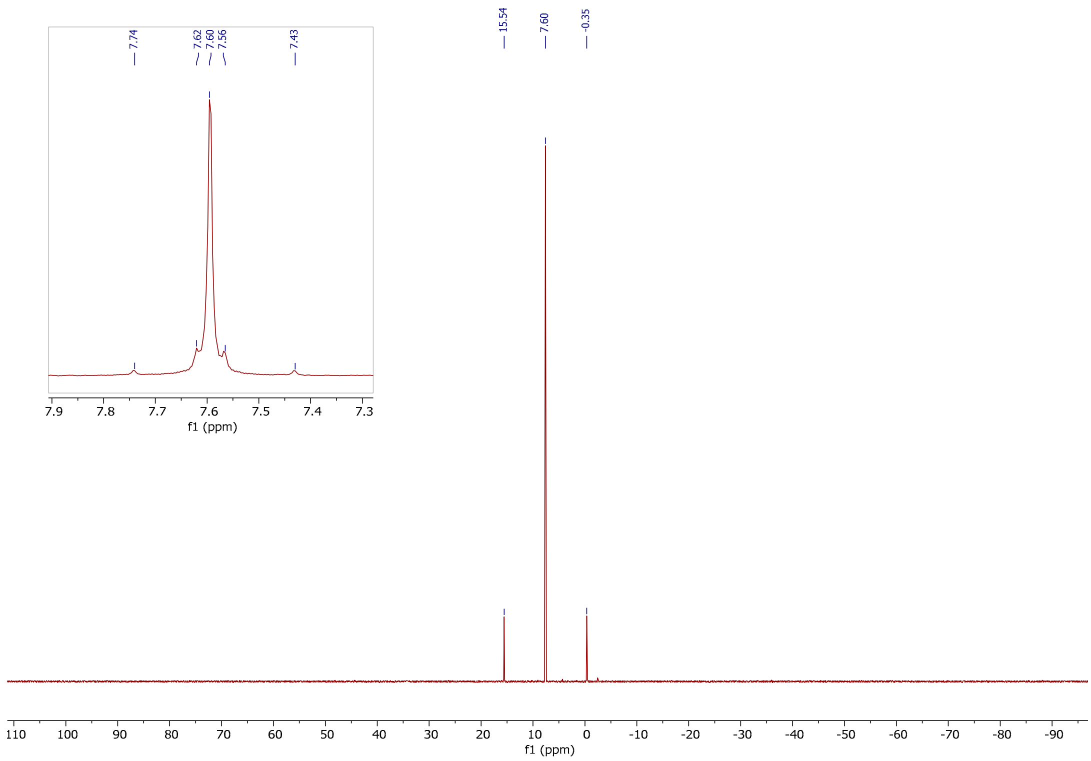

Figure S18. The $^{31}P\{^1H\}$ NMR spectra of $[PtI_2(CNCy)(PPh_3)_2]$ **6**.

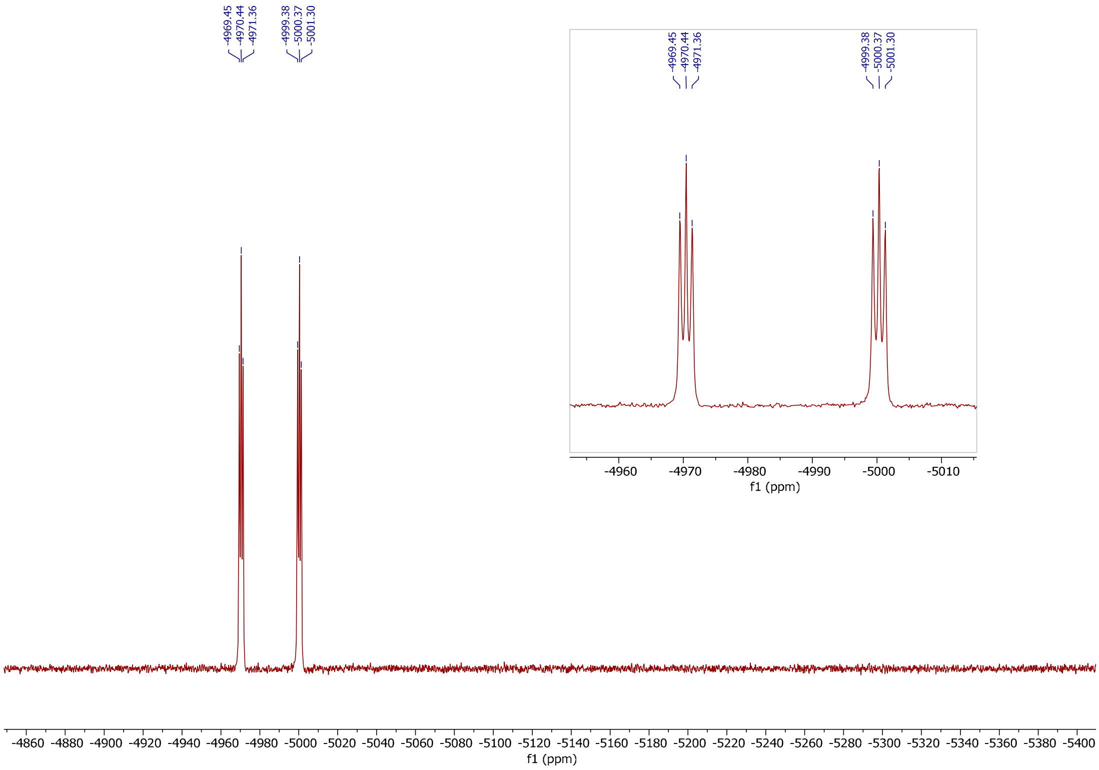

Figure S19. The $^{195}Pt\{^1H\}$ NMR spectra of $[PtI_2(CNCy)(PPh_3)_2]$ **6**.

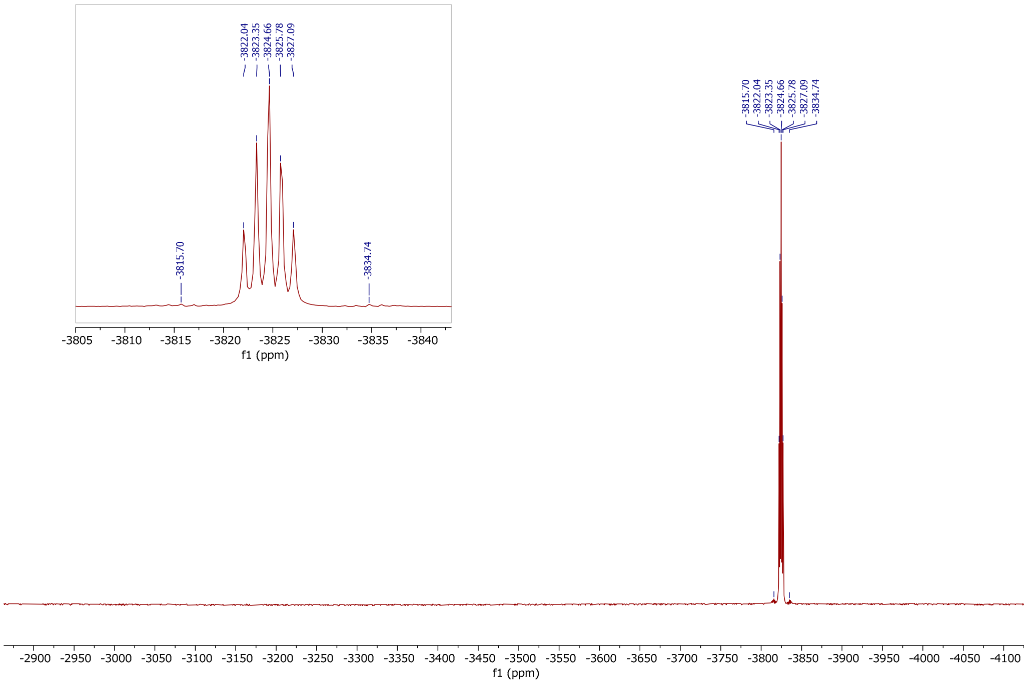

Figure S20. The $^{195}Pt\{^{1}H\}$ NMR spectra of $[PtCl_2(CNCy)_2]$ **7**.

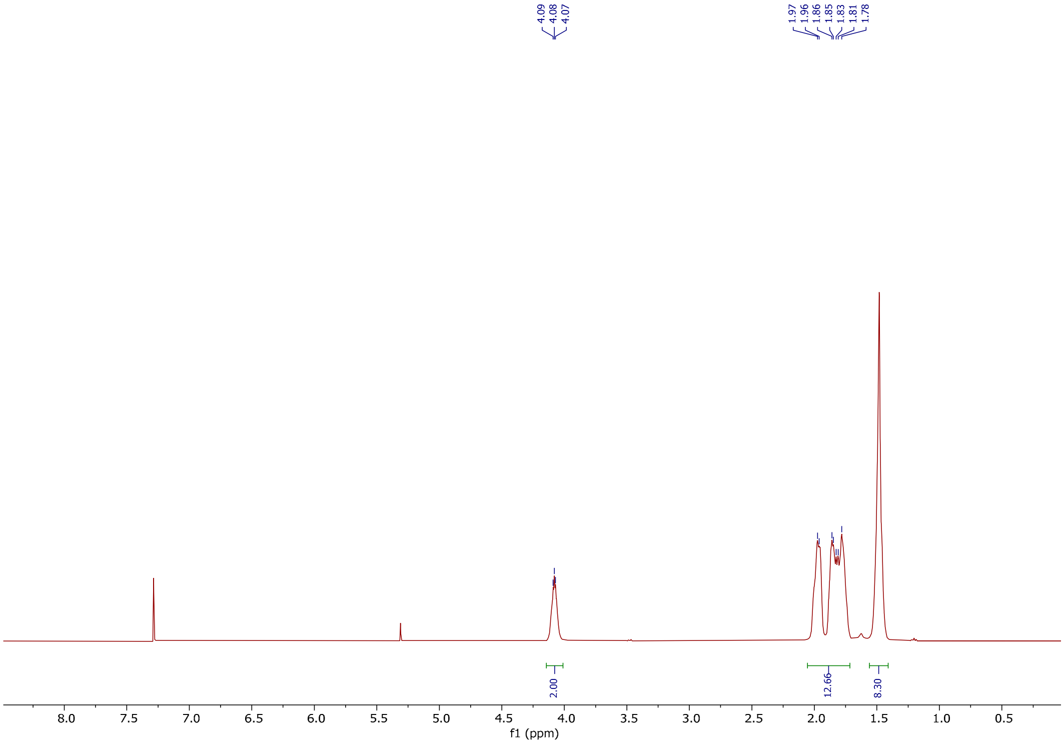

Figure S21. The ^{1}H NMR spectra of $[PtBr_2(CNCy)_2]$ **8**.

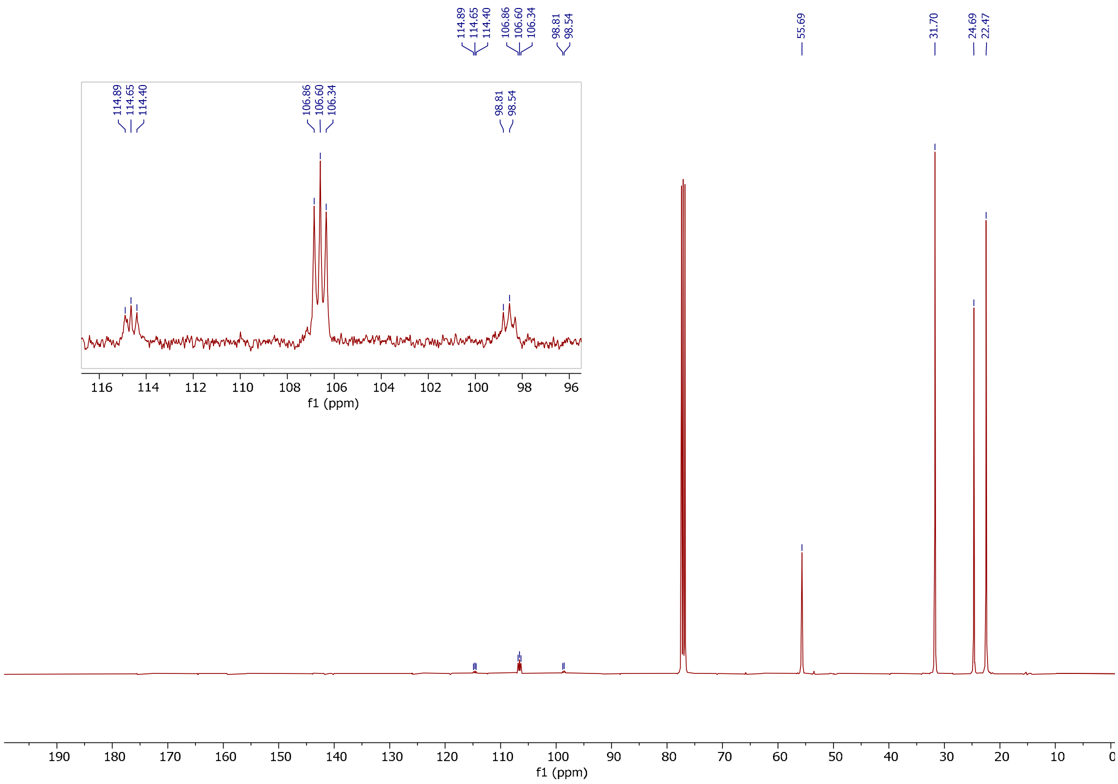

Figure S22. The $^{13}C\{^1H\}$ NMR spectra of $[PtBr_2(CNCy)_2]$ **8**.

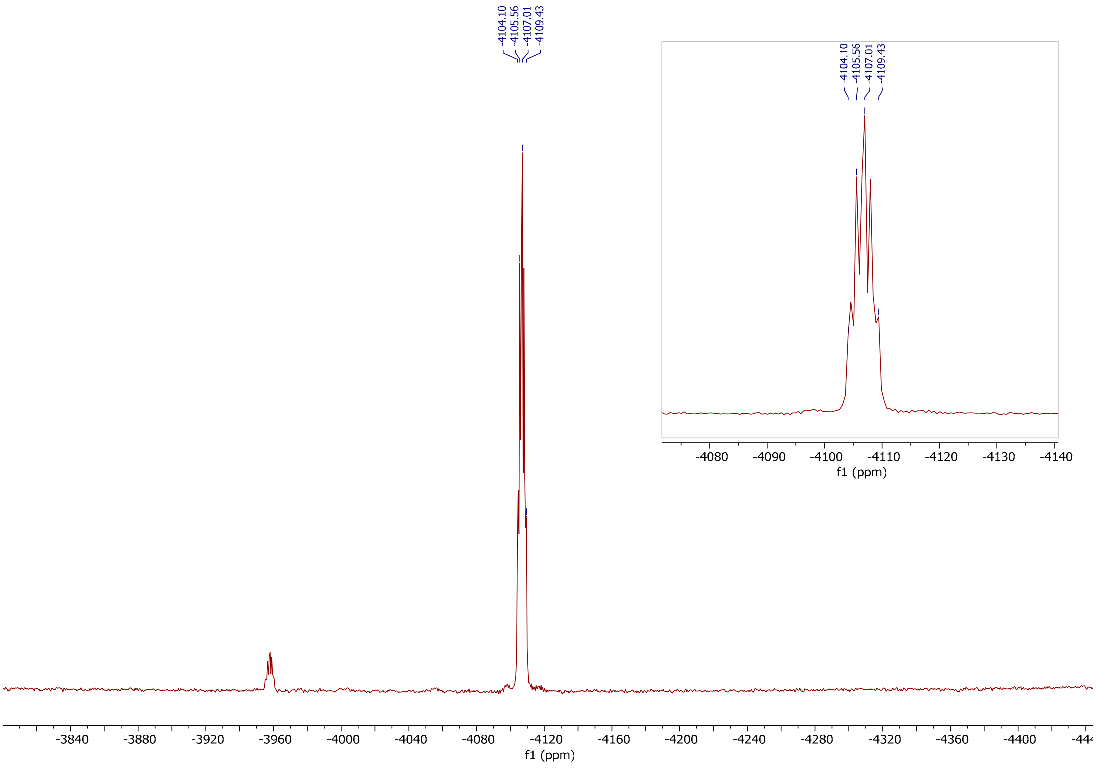

Figure S23. The $^{195}Pt\{^1H\}$ NMR spectra of $[PtBr_2(CNCy)_2]$ **8**.

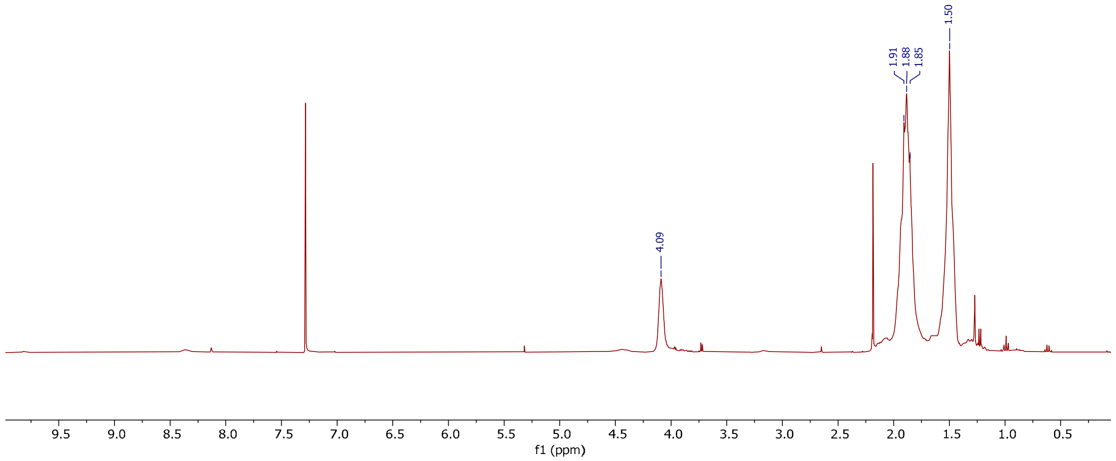

Figure S24. The ^{1}H NMR spectra of $[PtI_2(CNCy)_2]$ **9**.

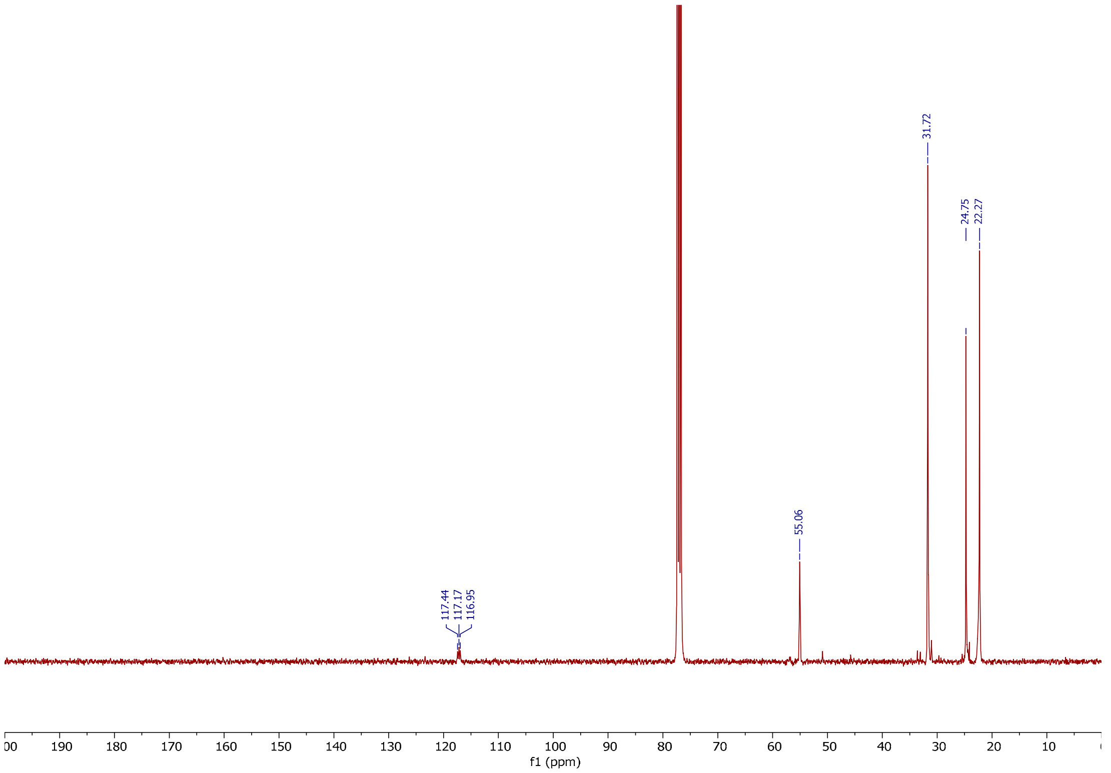

Figure S25. The $^{13}C\{^{1}H\}$ NMR spectra of $[PtI_2(CNCy)_2]$ **9**.

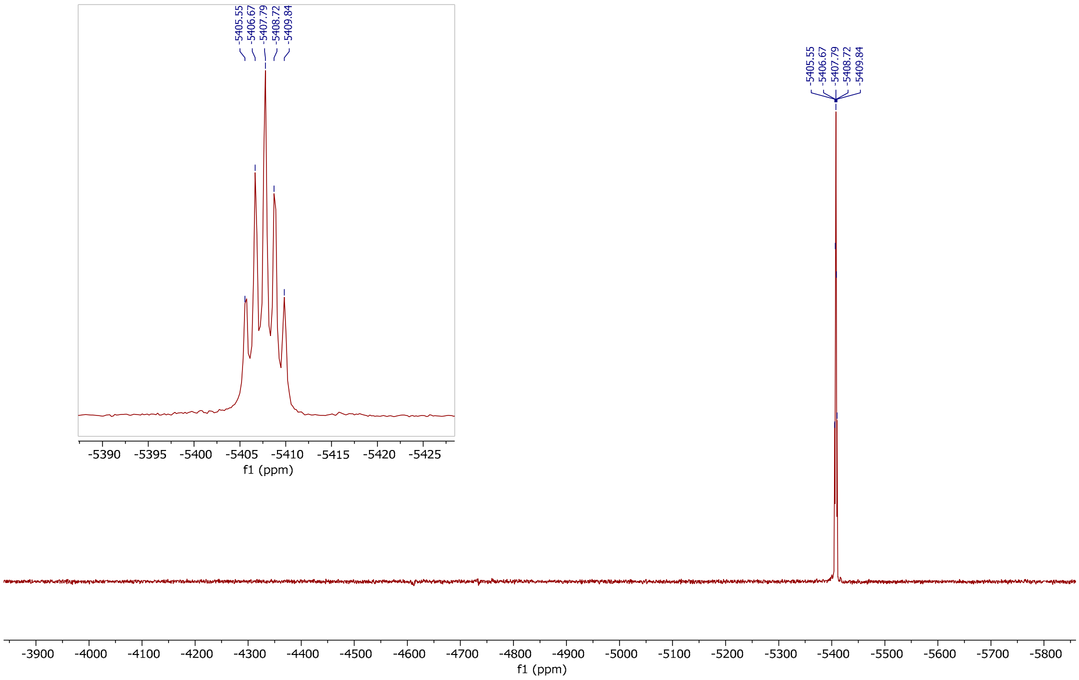

Figure S26. The $^{195}Pt\{^{1}H\}$ NMR spectra of $[PtI_2(CNCy)_2]$ **9**.

S8. FTIR spectra

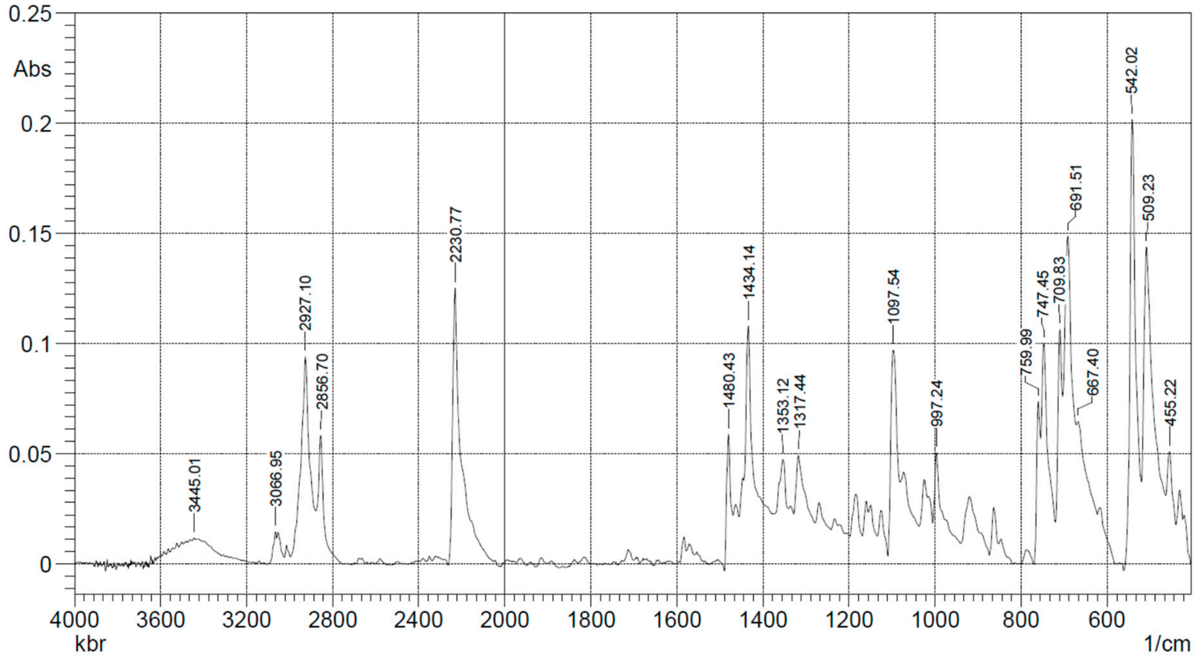

Figure S27. The FTIR spectra of $[PtCl_2(CNCy)(PPh_3)_2]$ **4**.

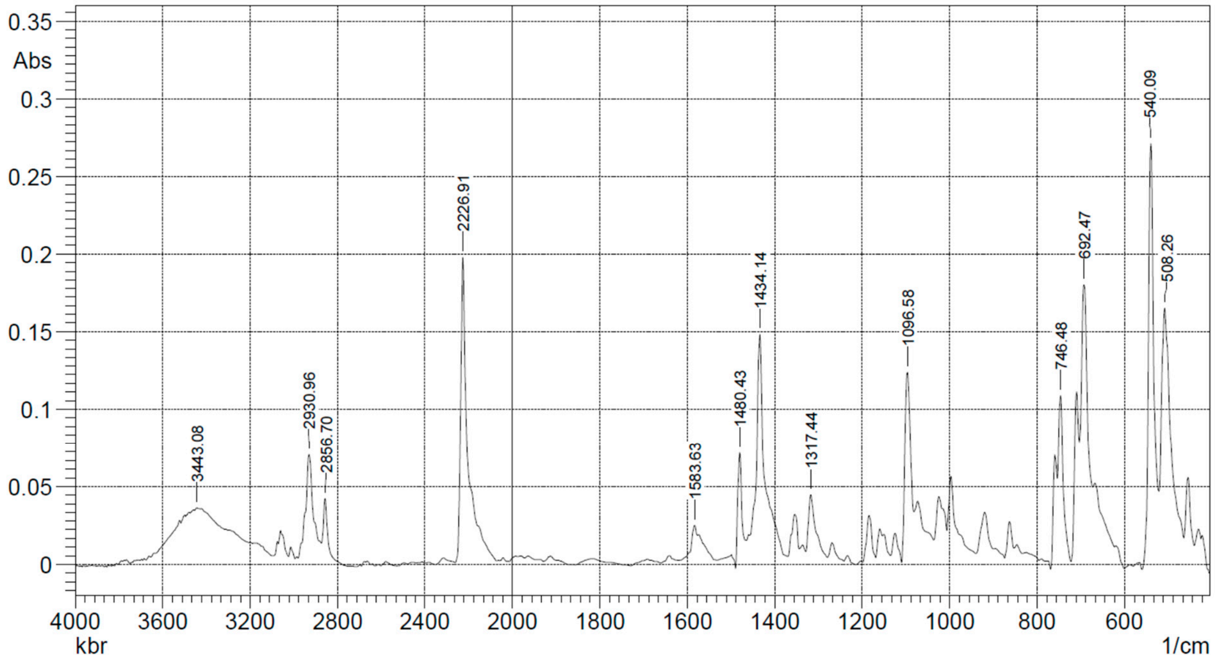

Figure S28. The FTIR spectra of $[PtBr_2(CNCy)(PPh_3)_2]$ **5**.

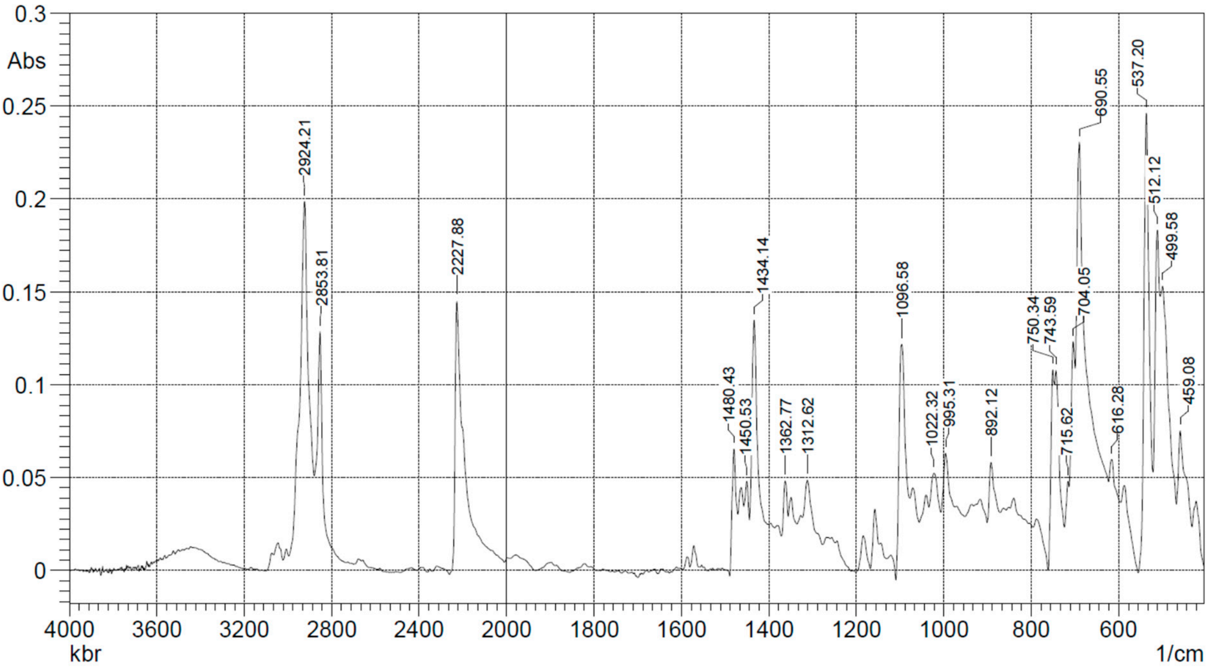

Figure S29. The FTIR spectra of $[PtI_2(CNCy)(PPh_3)_2]$ **6**.

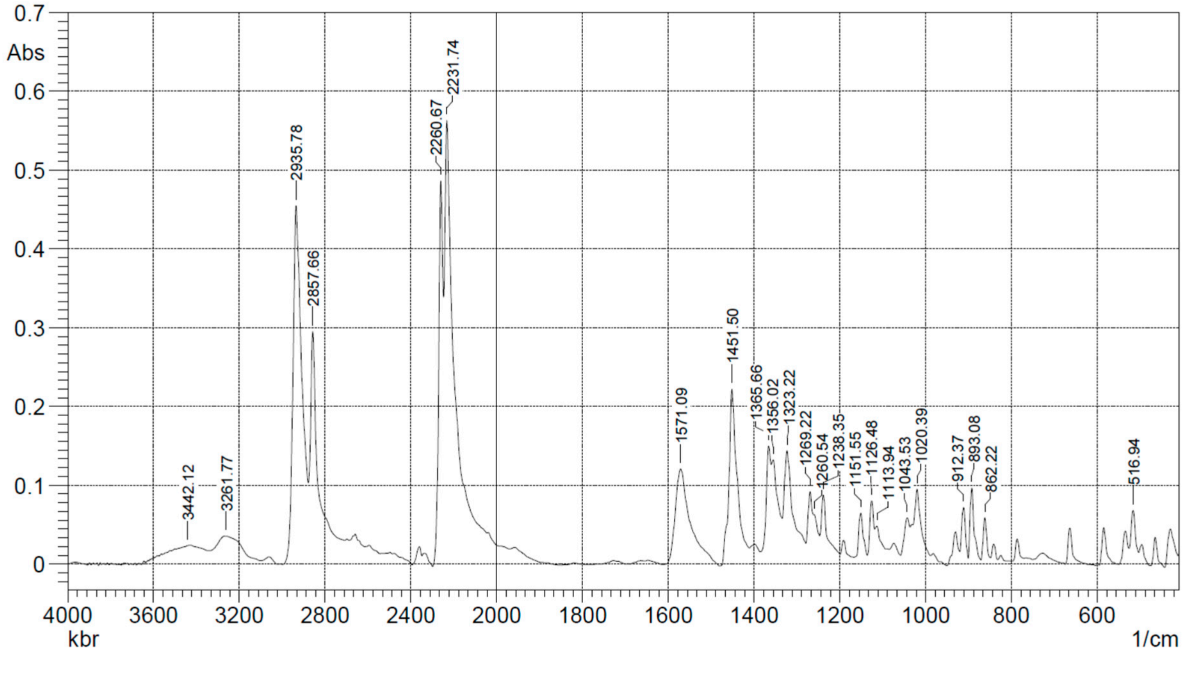

Figure S30. The FTIR spectra of $[PtBr_2(CNCy)_2]$ **8**.

S9. Mass spectra

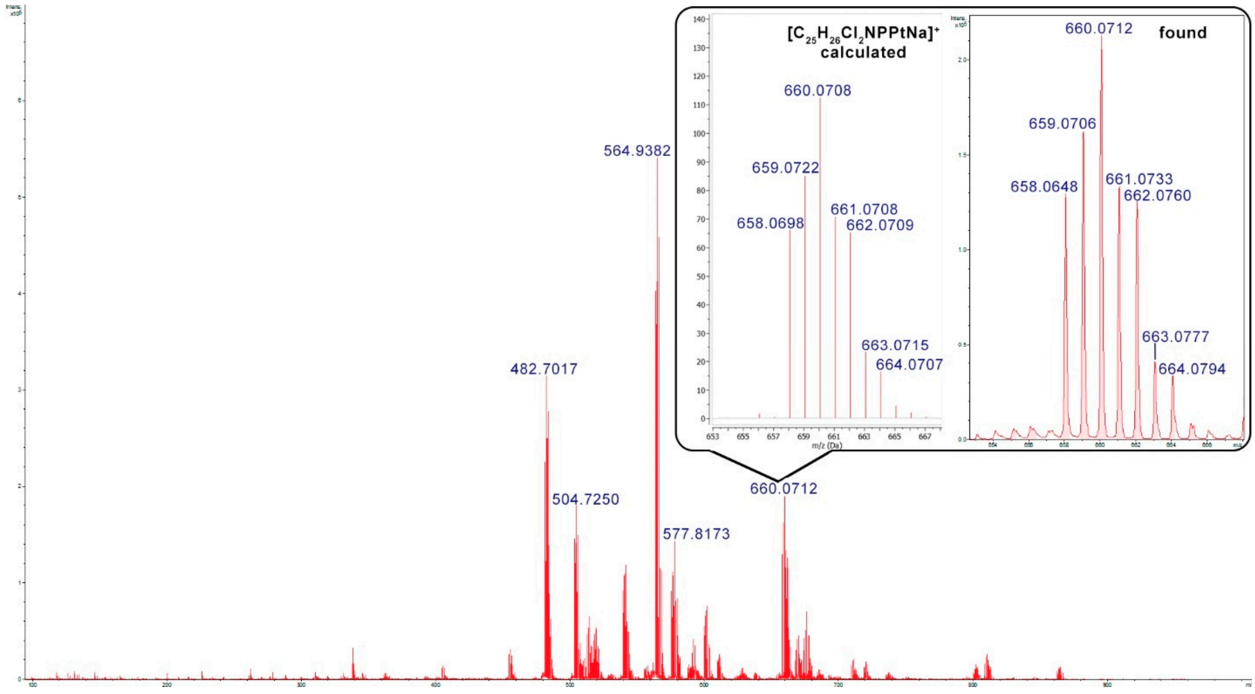

Figure S31. The mass spectra of [$PtCl_2(CNCy)(PPh_3)_2$] **4**.

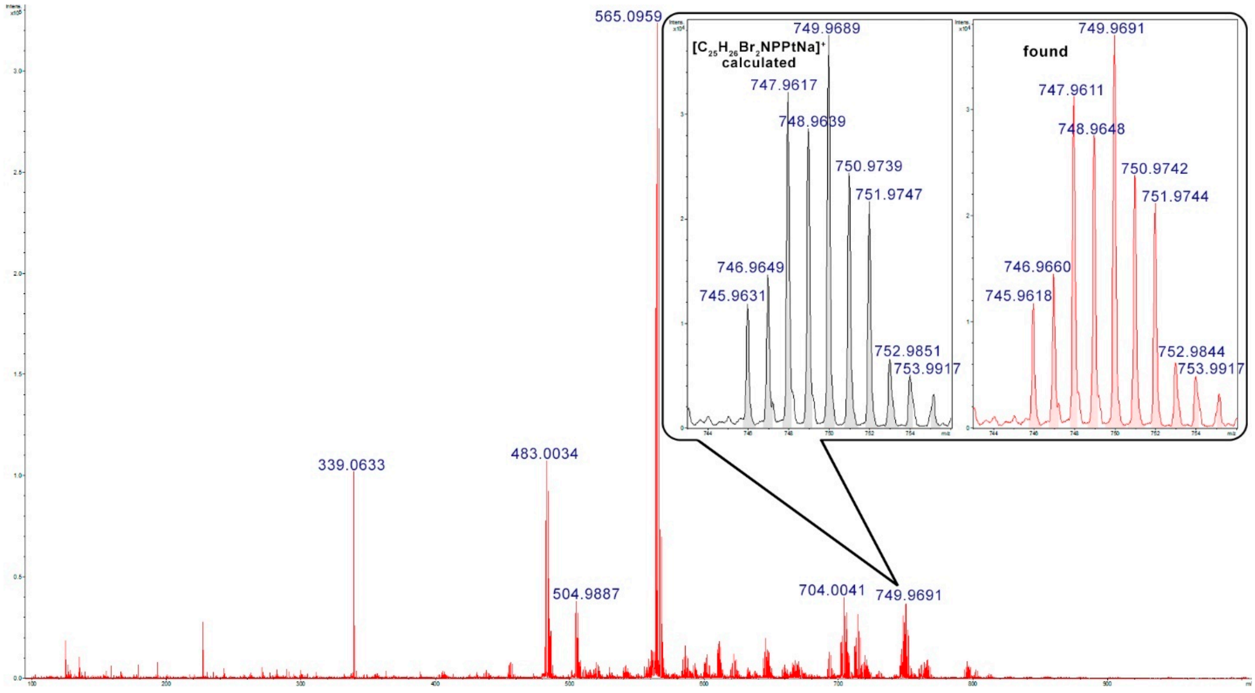

Figure S32. The mass spectra of [$PtBr_2(CNCy)(PPh_3)_2$] **5**.

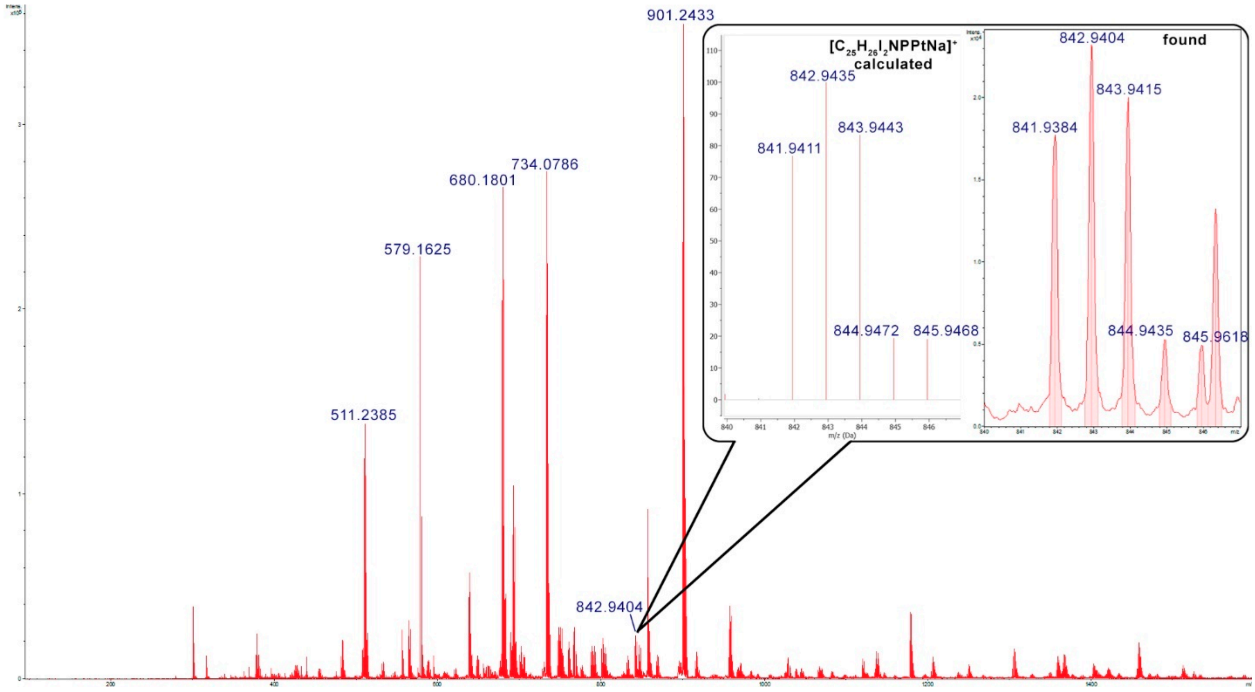

Figure S33. The mass spectra of [$PtI_2(CNCy)(PPh_3)_2$] **6**.

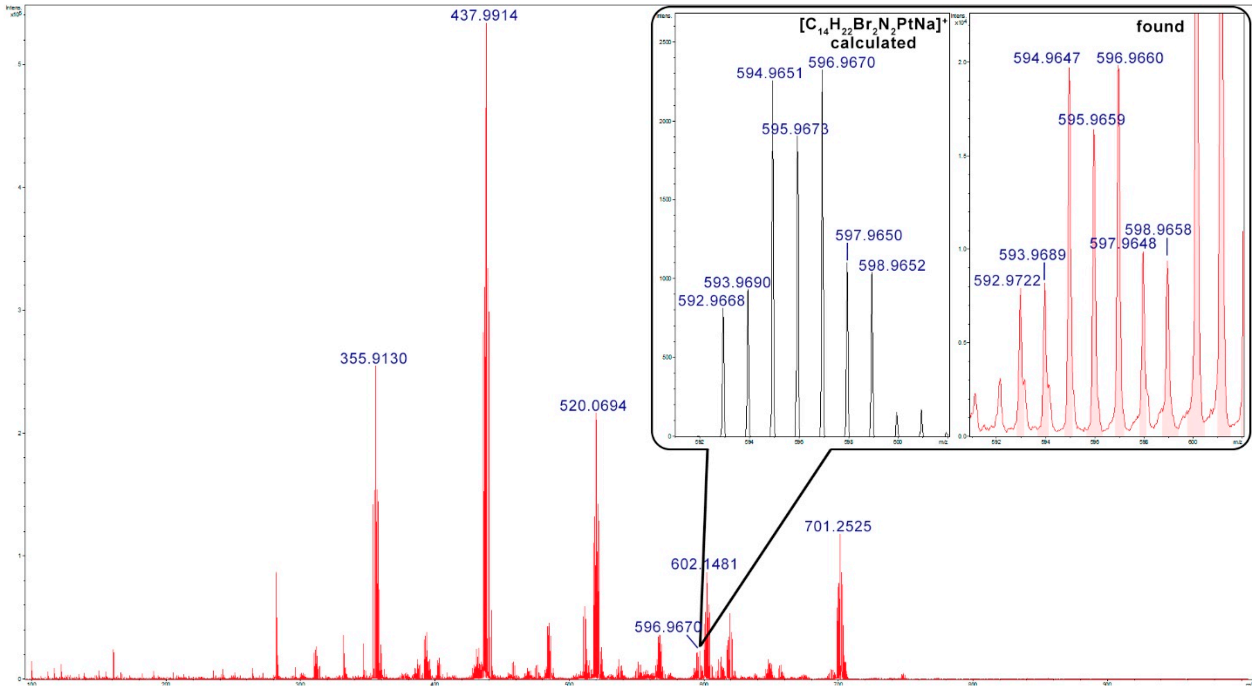

Figure S34. The mass spectra of [$PtBr_2(CNCy)_2$] **8**.

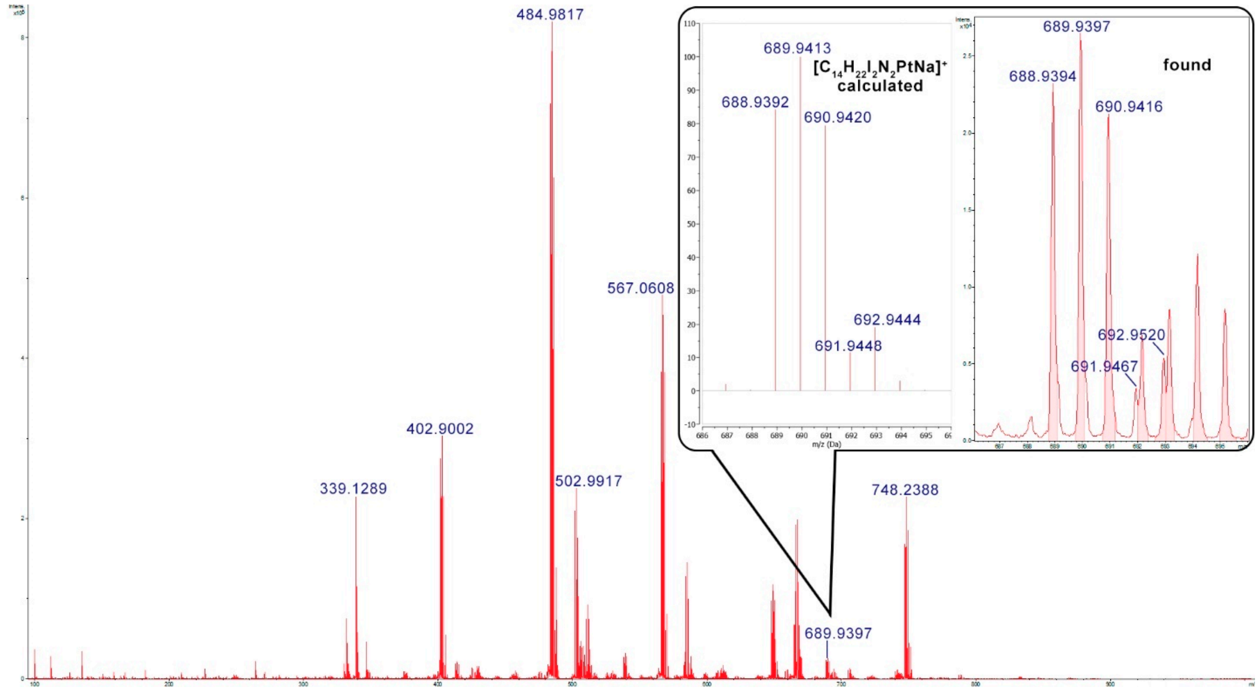

Figure S35. The mass spectra of [PtI$_2$(CNCy)$_2$] **9**.

References

1. Katkova, S.A., M.A. Kinzhalov, P.M. Tolstoy, A.S. Novikov, V.P. Boyarskiy, A.Y. Ananyan, P.V. Gushchin, M. Haukka, A.A. Zolotarev, A.Y. Ivanov, S.S. ZlotskyV.Y. Kukushkin, Diversity of Isomerization Patterns and Protolytic Forms in Aminocarbene PdII and PtII Complexes Formed upon Addition of N,N′-Diphenylguanidine to Metal-Activated Isocyanides. *Organometallics*, **2017**.
2. Tskhovrebov, A.G., K.V. Luzyanin, M. HaukkaV.Y. Kukushkin, Synthesis and Characterization of cis-(RNC)2PtII Species Useful as Synthons for Generation of Various (Aminocarbene)PtII Complexes. *Journal of Chemical Crystallography*, **2012**, 42, pp. 1170-1175.
3. Rigamonti, L., C. Manassero, M. Rusconi, M. ManasseroA. Pasini, cis Influence in trans-Pt(PPh3)2 complexes. *Dalton Trans.*, **2009**, pp. 1206–1213.
4. Rigamonti, L., A. Forni, M. Manassero, C. ManasseroA. Pasini, Cooperation between Cis and Trans Influences in cis-PtII(PPh3)2 Complexes: Structural, Spectroscopic, and Computational Studies. *Inorg. Chem.*, **2010**, 49, pp. 123–135.
5. Kinzhalov, M.A., M.V. Kashina, A.S. Mikherdov, S.A. KatkovaV.V. Suslonov, Synthesis of Platinum(II) Phoshine Isocyanide Complexes and Study of Their Stability in Isomerization and Ligand Disproportionation Reactions. *Russ. J. Gen. Chem.*, **2018**, 88, pp. 1180-1187.
6. Manojlovicmuir, L., K.W. MuirT. Solomun, CIS-INFLUENCE AND TRANS-INFLUENCE OF LIGANDS IN PLATINUM(II) COMPLEXES - CRYSTAL AND MOLECULAR-STRUCTURE OF CIS- PTCL2(PET3)(CO). *Journal of Organometallic Chemistry*, **1977**, 142, pp. 265-280.
7. Briggs, J.R., C. CrockerB.L. Shaw, COMPLEXES OF THE TYPE TRANS-PTCL2(PR3)(CNR') AND THE CARBENE COMPLEX TRANS- PTCL2(PR3) C(NHC6H4ME-4)2. *Inorganica Chimica Acta-Articles*, **1981**, 51, pp. 15-18.
8. Kinzhalov, M.A., M.V. Kashina, A.S. Mikherdov, E.A. Mozheeva, A.S. Novikov, A.S. Smirnov, D.M. Ivanov, M.A. Kryukova, A.Y. Ivanov, S.N. Smirnov, V.Y. KukushkinK.V. Luzyanin, Dramatically Enhanced Solubility of Halide-Containing Organometallic Species in Diiodomethane: The Role of Solvent···Complex Halogen Bonding. *Angew. Chem. Int. Ed.*, **2018**, 57, pp. 12785–12789.
9. Zhang, B., K. KurpiewskaA. Dömling, Highly Stereoselective Ugi/Pictet–Spengler Sequence. *The Journal of Organic Chemistry*, **2022**, 87, pp. 7085-7096.
10. Katkova, S.A., K.V. Luzyanin, A.S. NovikovM.A. Kinzhalov, Modulation of luminescence properties for [cyclometalated]-PtII(isocyanide) complexes upon co-crystallisation with halosubstituted perfluorinated arenes. *New J. Chem.*, **2021**, 45, pp. 2948-2952.
11. Lu, Z.-l., A. MayrK.-K. Cheung, Synthesis and formation of metal complexes of 4-alkynyl and 4-cyano-2,6-diisopropylphenylisocyanides. *Inorganica Chimica Acta*, **1999**, 284, pp. 205-214.
12. Sokolova, E.V., M.A. Kinzhalov, A.S. Smirnov, A.M. Cheranyova, D.M. Ivanov, V.Y. KukushkinN.A. Bokach, Polymorph-Dependent Phosphorescence of Cyclometalated Platinum(II) Complexes and Its Relation to Non-covalent Interactions. *ACS Omega*, **2022**, 7, pp. 34454-34462.
13. Stephany, R.W., M.J.A. de BieW. Drenth, A 13C-NMR and IR study of isocyanides and some of their complexes. *Organic Magnetic Resonance*, **1974**, 6, pp. 45–47.

14. Kinzhalov, M.A., K.V. Luzyanin, I.A. Boyarskaya, G.L. StarovaV.P. Boyarskiy, Synthetic and structural investigation of PdBr2(CNR)(2) (R = Cy, Xyl). *Journal of Molecular Structure*, **2014**, 1068, pp. 222-227.
15. Knorr, M., I. Jourdain, G. Crini, K. Frank, H. SachdevC. Strohmann, Synthesis, Reactivity and Molecular Structures of Bis(diphenylphosphanyl)methane-Bridged Heterobimetallic Iron−Platinum Isocyanide Complexes: Breaking and Formation of Metal−Metal Bonds. *European Journal of Inorganic Chemistry*, **2002**, 2002, pp. 2419-2426.
16. Vicenzi, D., P. Sgarbossa, A. Biffis, C. Tubaro, M. Basato, R.A. Michelin, A. Lanza, F. Nestola, S. Bogialli, P. PastoreA. Venzo, Platinum(II) Complexes with Novel Diisocyanide Ligands: Catalysts in Alkyne Hydroarylation. *Organometallics*, **2013**, 32, pp. 7153-7162.
17. Kim, Y.-J., E.-H. ChoiS.W. Lee, Reactivity of Bis(silyl) Platinum(II) Complexes toward Isocyanides: Preparation and Structure of cis-[Pt(SiHPh2)2(CNR)(PR′3)] (R = t-Bu, cyclohexyl, i-Pr; R′ = Me, Et). *Organometallics*, **2003**, 22, pp. 3316-3319.
18. Jameson, C.J., in *Multinuclear NMR*, J. Mason, Editor. 1987, Plenum Press: New York. p. 89–131.
19. Pidcock, A., R.E. RichardsL.M. Venanzi, 195Pt-31P nuclear spin coupling constants and the nature of the trans-effect in platinum complexes. *J. Chem. Soc. A*, **1966**, pp. 1707-1710.
20. Sluch, I.M., A.J. MirandaL.M. Slaughter, Channeled Polymorphs of cis-M(CNPh)2Cl2 (M = Pt, Pd) With Extended Metallophilic Interactions. *Crystal Growth & Design*, **2009**, 9, pp. 1267-1270.
21. Kashina, M.V., D.M. IvanovM.A. Kinzhalov, The Isocyanide Complexes cis-[MCl2(CNC6H4-4-X)2] (M = Pd, Pt; X = Cl, Br) as Tectons in Crystal Engineering Involving Halogen Bonds. *Crystals*, **2021**, 11, pp. 799.
22. Harvey, P.D., K.D. Truong, K.T. Aye, M. DrouinA.D. Bandrauk, Resonance-Enhanced Intraligand and Metal-Metal Raman Modes in Weakly Metal-Metal-Interacting Platinum(II) Complexes and Long-Range Relationship between Metal-Metal Separations and Force Constants. *Inorganic Chemistry*, **1994**, 33, pp. 2347-2354.
23. Barnett, B.R., C.E. Moore, A.L. RheingoldJ.S. Figueroa, Cooperative Transition Metal/Lewis Acid Bond-Activation Reactions by a Bidentate (Boryl)iminomethane Complex: A Significant Metal–Borane Interaction Promoted by a Small Bite-Angle LZ Chelate. *Journal of the American Chemical Society*, **2014**, 136, pp. 10262-10265.
24. Sluch, I.M., A.J. Miranda, O. Elbjeirami, M.A. OmaryL.M. Slaughter, Interplay of Metallophilic Interactions, π–π Stacking, and Ligand Substituent Effects in the Structures and Luminescence Properties of Neutral PtII and PdII Aryl Isocyanide Complexes. *Inorg. Chem.*, **2012**, 51, pp. 10728-10746.
25. Kinzhalov, M.A., M.V. Kashina, A.S. Mikherdov, S.A. KatkovaV.V. Suslonov, Synthesis of Platinum(II) Phoshine Isocyanide Complexes and Study of Their Stability in Isomerization and Ligand Disproportionation Reactions. *Russian Journal of General Chemistry*, **2018**, 88, pp. 1180-1187.
26. Vicente, J., A. Arcas, J.M. Fernández-Hernández, G. AullónD. Bautista, Acetonyl Platinum(II) Complexes. *Organometallics*, **2007**, 26, pp. 6155-6169.
27. Hubbert, C., M. Breunig, K.J. Carroll, F. RomingerA.S.K. Hashmi, Simple Synthesis of New Mixed Isocyanide-NHC-Platinum(II) Complexes and Their Catalytic Activity. *Australian Journal of Chemistry*, **2014**, 67, pp. 469-469.
28. Bulatova, M., D.M. Ivanov, J.M. Rautiainen, M.A. Kinzhalov, K.-N. Truong, M. LahtinenM. Haukka, Studies of Nature of Uncommon Bifurcated I–I···(I–M) Metal-Involving Noncovalent Interaction in Palladium(II) and Platinum(II) Isocyanide Cocrystals. *Inorg. Chem.*, **2021**, 60, pp. 13200–13211.

29. Mayr, A., S. Wang, K.-K. CheungM. Hong, Synthesis, structure, and liquid crystal properties of a series of platinum(II) complexes containing chiral 4-(4-alkoxyphenylethynyl) phenylisocyanide ligands. *Journal of Organometallic Chemistry*, **2003**, 684, pp. 287-299.
30. Wang, S., A. MayrK.-k. Cheung, Mesogenic palladium and platinum-diiodide complexes of4-isocyanobenzylidene-4-alkoxyphenylimines. *Journal of Materials Chemistry*, **1998**, 8, pp. 1561-1565.
31. Kashina, M.V., M.A. Kinzhalov, A.S. Smirnov, D.M. Ivanov, A.S. NovikovV.Y. Kukushkin, Dihalomethanes as Bent Bifunctional XB/XB-Donating Building Blocks for Construction of Metal-involving Halogen Bonded Hexagons. *Chem.: Asian J.*, **2019**, 14, pp. 3915-3920.
32. Kaharu, T., T. Tanaka, M. SawadaS. Takahashi, Liquid-crystalline palladium– and platinum–isonitrile complexes: synthesis, mesomorphic properties and molecular structure. *J. Mater. Chem.*, **1994**, 4, pp. 859-865.
33. Yamamoto, Y., T. HagiwaraH. Yamazaki, Axially dissymmetric 2,2′-diisocyano-1,1′-binaphthyl and its platinum complexes. *Inorganica Chimica Acta*, **1986**, 115, pp. L35-L37.
